# Supplementary material for: Role of 3D printing technology in paediatric teaching and training: a systematic review
Source: BMJ Paediatr Open. 2021 Dec 7;5(1):e001050. doi: 10.1136/bmjpo-2021-001050 (PMC8655595; doi:10.1136/bmjpo-2021-001050)
Supplement: Author's manuscript [file bmjpo-2021-001050.draft_revisions.pdf]

BMJ Paediatrics Open

BMJ Paediatrics Open is committed to open peer review. As part of this commitment we make the peer review history of every article we publish publicly available.

When an article is published we post the peer reviewers' comments and the authors' responses online. We also post the versions of the paper that were used during peer review. These are the versions that the peer review comments apply to.

The versions of the paper that follow are the versions that were submitted during the peer review process. They are not the versions of record or the final published versions. They should not be cited or distributed as the published version of this manuscript.

BMJ Paediatrics Open is an open access journal and the full, final, typeset and author-corrected version of record of the manuscript is available on our site with no access controls, subscription charges or pay-per-view fees (http://bmjpaedsopen.bmj.com).

If you have any questions on BMJ Paediatrics Open's open peer review process please email info.bmjpo@bmj.com

BMJ Paediatrics Open

The Role of 3D Printing Technology in Paediatric Teaching and Training: a Systematic Review

Journal:	*BMJ Paediatrics Open*
Manuscript ID	bmjpo-2021-001050
Article Type:	Original research
Date Submitted by the Author:	05-Feb-2021
Complete List of Authors:	Asif, Ashar; University of Bristol Faculty of Medicine and Dentistry, Lee, Elgin; Newcastle Upon Tyne Hospitals NHS Foundation Trust, Caputo, Massimo; University of Bristol Faculty of Medicine and Dentistry; University of Bristol School of Clinical Science, Bristol Heart Institute Biglino, Giovanni; University of Bristol School of Clinical Science, Bristol Heart Institute; Imperial College London National Heart and Lung Institute Shearn, Andrew; University of Bristol Faculty of Medicine and Dentistry; University of Bristol School of Clinical Science, Bristol Heart Institute
Keywords:	Data Collection, Information Technology, Technology, Cardiology, Neurosurgery

SCHOLARONE™
Manuscripts

I, the Submitting Author has the right to grant and does grant on behalf of all authors of the Work (as defined in the below author licence), an exclusive licence and/or a non-exclusive licence for contributions from authors who are: i) UK Crown employees; ii) where BMJ has agreed a CC-BY licence shall apply, and/or iii) in accordance with the terms applicable for US Federal Government officers or employees acting as part of their official duties; on a worldwide, perpetual, irrevocable, royalty-free basis to BMJ Publishing Group Ltd ("BMJ") its licensees and where the relevant Journal is co-owned by BMJ to the co-owners of the Journal, to publish the Work in this journal and any other BMJ products and to exploit all rights, as set out in our licence.

The Submitting Author accepts and understands that any supply made under these terms is made by BMJ to the Submitting Author unless you are acting as an employee on behalf of your employer or a postgraduate student of an affiliated institution which is paying any applicable article publishing charge ("APC") for Open Access articles. Where the Submitting Author wishes to make the Work available on an Open Access basis (and intends to pay the relevant APC), the terms of reuse of such Open Access shall be governed by a Creative Commons licence – details of these licences and which Creative Commons licence will apply to this Work are set out in our licence referred to above.

Other than as permitted in any relevant BMJ Author's Self Archiving Policies, I confirm this Work has not been accepted for publication elsewhere, is not being considered for publication elsewhere and does not duplicate material already published. I confirm all authors consent to publication of this Work and authorise the granting of this licence.

Confidential: For Review Only

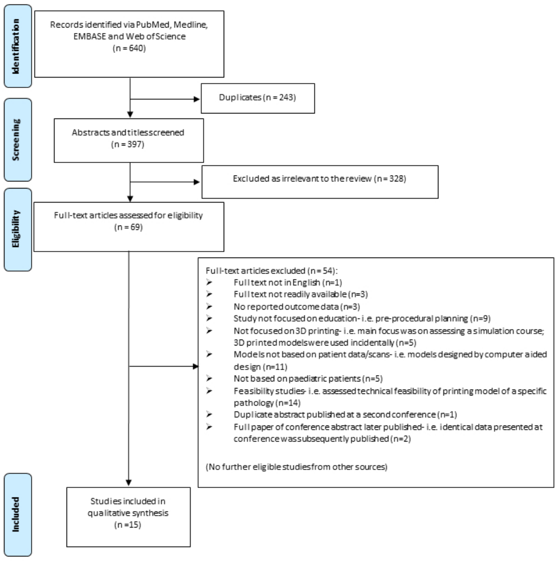
Identification
Records identified via PubMed, Medline, EMBASE and Web of Science
(n = 640)
Duplicates (n = 243)
Screening
Abstracts and titles screened
(n = 397)
Excluded as irrelevant to the review (n = 328)
Eligibility
Full-text articles assessed for eligibility
(n = 69)
Full-text articles excluded (n = 54):
Full text not in English (n=1)
Full text not readily available (n=3)
No reported outcome data (n=3)
Study not focused on education- i.e. pre-procedural planning (n=9)
Not focused on 3D printing- i.e. main focus was on assessing a simulation course; 3D printed models were used incidentally (n=5)
Models not based on patient data/scans- i.e. models designed by computer aided design (n=11)
Not based on paediatric patients (n=5)
Feasibility studies- i.e. assessed technical feasibility of printing model of a specific pathology (n=14)
Duplicate abstract published at a second conference (n=1)
Full paper of conference abstract later published- i.e. identical data presented at conference was subsequently published (n=2)
(No further eligible studies from other sources)
Included
Studies included in qualitative synthesis
(n =15)

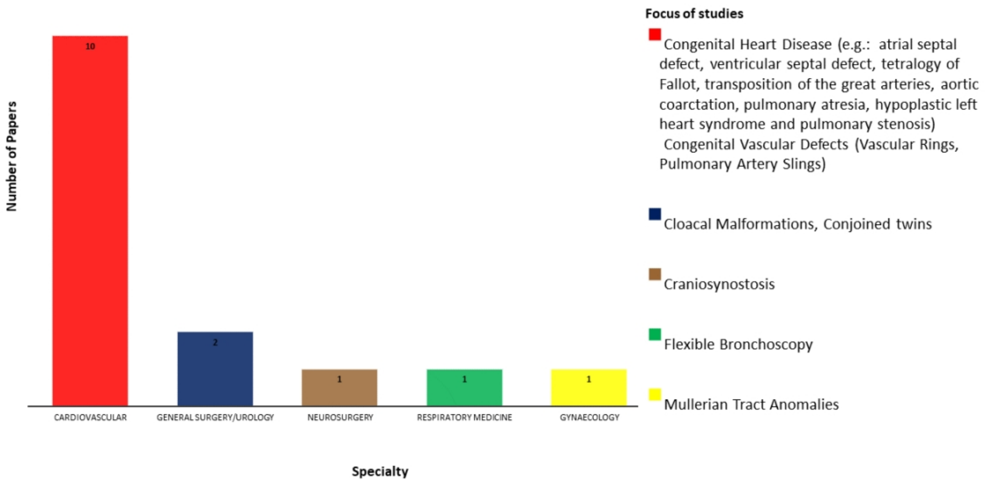
Focus of studies
Congenital Heart Disease (e.g.: atrial septal defect, ventricular septal defect, tetralogy of Fallot, transposition of the great arteries, aortic coarctation, pulmonary atresia, hypoplastic left heart syndrome and pulmonary stenosis)
Congenital Vascular Defects (Vascular Rings, Pulmonary Artery Slings)
Cloacal Malformations, Conjoined twins
Craniosynostosis
Flexible Bronchoscopy
Mullerian Tract Anomalies
Number of Papers
10
2
1
1
1
CARDIOVASCULAR
GENERAL SURGERY/UROLOGY
NEUROSURGERY
RESPIRATORY MEDICINE
GYNAECOLOGY
Specialty

The Role of 3D Printing Technology in Paediatric Teaching and Training: a Systematic Review

Ashar Asif[1], ORCiD 0000-0002-9361-1083

Elgin Meng Lee[2] ORCiD 0000-0002-0054-1574

Massimo Caputo[1, 3] ORCiD 0000-0001-7508-0891

Giovanni Biglino[3, 4] ORCiD 0000-0003-0413-149X

Andrew I. U. Shearn[1, 3] ORCiD 0000-0002-3741-9118

1. Bristol Medical School, University of Bristol, Bristol Royal Infirmary, Bristol, UK, BS2 8HW,

2. Newcastle upon Tyne Hospitals NHS Foundation Trust, Freeman Road, Newcastle upon Tyne, UK, NE7 7DN

3. Bristol Heart Institute, Bristol Royal Infirmary, Bristol, UK, BS2 8HW

4. National Heart and Lung Institute, Guy Scadding Building, Imperial College London, London, UK, SW3 6LY

Corresponding author

Dr Andrew Shearn

University of Bristol

Research Floor Level 7

Bristol Royal Infirmary

Upper Maudlin Street

Bristol BS2 8HW

Tel: (44) 0117 3423142

Email: andrew.shearn@bristol.ac.uk

Word count: 2499

Confidential: For Review Only

Abstract

Background

In the United Kingdom, undergraduate paediatric training is brief resulting in trainees with a lower paediatric knowledge base compared to other aspects of medicine. With congenital conditions being successfully treated at childhood, adult clinicians encounter and will need to understand more complex pathology. Patient-specific 3D printed (3DP) models have been used in clinical training, especially for rarer, complex conditions. We perform a systematic review to evaluate the evidence base in using 3DP models to train paediatric trainees, clinicians and medical students.

Methods

Online databases PubMed, Web of Science, EMBASE and Ovid Medline were searched between January 2010 and April 2020 using search terms relevant to “paediatrics”, “education”, “training” and “3D printing”. Participants were medical students, postgraduate trainees or clinical staff. Comparative studies (patient-specific 3DP models vs. traditional teaching methods) and non-comparative studies were included. Outcomes gauged objective and subjective measures; test scores, time taken to complete tasks, self-reported confidence and personal preferences on 3DP models. If reported, the cost of and time taken to produce the models were noted.

Results

From 640 results, 15 studies fit the criteria of the review protocol. Participants using 3DP models had greater improvement in test scores and faster times to complete procedures and identify anatomical landmarks compared to traditional teaching methods (2D diagrams, lectures, videos, supervised clinical events). User feedback was positive, reporting greater user self-confidence in understanding concepts with users wishing for integrated use of 3DP in regular teaching. Four studies reported the costs and times of production, which varied depending on model complexity and printer. 3DP

Confidential: For Review Only

models were cheaper than "off-the-shelf" models available on the market and with the benefit of utilising real-world pathologies. These mostly non-randomised and single-centred studies did not address bias or report long-term and clinically translatable outcomes.

Conclusions

3DP models are associated with greater user satisfaction and good short-term educational outcomes, with low quality evidence. Multicentred, randomised studies with long-term follow-up and clinically assessed outcomes are needed to fully assess the benefits in this setting.

Systematic review registration

The review is registered on Prospero. Prospero ID: CRD42020179656

Available from: https://www.crd.york.ac.uk/prospero/display_record.php?ID=CRD42020179656

What is known about the subject

- Undergraduate paediatric education is known to be short and postgraduate paediatric training can be limited within certain regions
- Patients with congenital diseases are surviving to adulthood, meaning adult clinicians are seeing an increase of cases which were traditionally considered as 'paediatric conditions'
- Patient specific 3D printed models have been used in other medical and surgical specialties for hands-on education and training and can compensate for these pitfalls

What this review adds

- 3D printed models in paediatric education and training sessions have been shown to improve immediate educational and procedural performance, with high user satisfaction
- The quality of evidence is poor due to unaddressed confounding factors, small study cohorts and poor study design

Confidential: For Review Only

- In the few studies that reported costs, 3D printed models had the benefit of producing models at relatively lower costs compared to alternative resources

Introduction

In 2017, the Royal College of Paediatrics and Child Health published a response to the “Shape of Training Review” of postgraduate training within the United Kingdom. They observed that “doctors coming into paediatric specialty training do so from a lower knowledge base than adult medicine” (1). This could partly be explained by the brevity of undergraduate exposure to paediatrics, typically 4-8 weeks in total (2). Furthermore some paediatric trainees report a lack of educational opportunities within their postgraduate training programme (3), potentially driven by the varied facilities offered by different regions and centres (4,5).

It is increasingly important for non-paediatric clinicians to fully appreciate paediatric pathologies. Novel therapies have allowed many children, who would have had poor outcomes from previously life-limiting diseases, to now live with such pathologies resulting in complex patients with chronic conditions (5). Clinical care and observation over the adult lifetime of these patients is often overseen by a myriad of clinicians, frequently requiring specialised oversight such as in the case of pregnant women with congenital heart disease (CHD) (6).

In recent years, 3D printing (3DP) has played a role in a variety of medical and surgical training and educational settings (7–9). 3DP models can replicate in-vivo clinical pathologies based on radiological images, allowing trainees to be exposed to pathologies and potential procedures they would not ordinarily experience through their routine clinical exposure (10). We aim to evaluate the current evidence base regarding the utility of 3DP compared to traditional teaching methods (i.e. 2D images/diagrams, lectures, videos, ward-based teaching) in educating and training of medical students and clinical trainees in paediatrics.

Confidential: For Review Only

Methods

Study Protocol

The study protocol is registered on PROSPERO and can be found with the following ID: CRD42020179656. Throughout the review, the authors referred and adhered to the Preferred Reporting Items for Systematic reviews and Meta-Analyses (PRISMA) guidelines (11).

Literature search strategy

The databases PubMed, Web of Science, EMBASE and Ovid Medline were searched. The search included all published material since January 2010 (last accessed in April 2020) in the English language. This timeframe was selected to ensure the studies reviewed used the most recent available technology. The following terms were truncated and combined using symbols (*) and Boolean operators (AND and OR) to maximise search results: 3D printing, paediatrics, education, teaching, training, session, and simulation. Synonyms and spelling variations were also searched to allow for international spellings and alternative terms (e.g. paediatrics vs. pediatrics).

Selection criteria

The inclusion criteria for final review were studies using 3DP models derived from paediatric patient imaging data (e.g. computed tomography (CT), magnetic resonance imaging (MRI), ultrasound (US) or echocardiography) and that occurred in an educational or training setting. This was defined as a pre-planned session or programme as part of a syllabus or as a stand-alone course, separate from a clinical setting and not aimed to directly contribute towards patient outcomes. This excluded studies which used 3DP models to facilitate communication with patients, carers or clinical staff. Studies that produced a 3DP model for pre-operative planning for a specific case were also excluded. Included studies evaluated the use of patient-specific models with undergraduate medical students or paediatric clinical staff. Comparative studies would compare the use of 3DP models against

Confidential: For Review Only

traditional educational methods such as lectures, tutorials, practical sessions, or textbooks. Case reports and case series were also included if they used the models in an educational setting and reported educational outcome measures. Published conference abstracts were included as per PRISMA guidance to minimise publication bias (12). Primary outcome measures were either objective measures such as pre-/post-intervention testing scores or procedure performance, or subjective measures such as participant reported opinions from questionnaires. Additional outcome measures that were recorded included the time taken to produce models and the cost of model production.

Data extraction and appraisal of evidence

The titles and abstracts of all papers obtained during the searches were screened for relevance by two authors independently (AA, EL). Relevant papers were then independently assessed for eligibility in full against the study protocol (AA, EL). Any disagreements were resolved by consensus, with the senior author (AS) acting as a final adjudicator for any unresolved discrepancies. The final included papers were then reviewed and appraised by AA and EL. Data was extracted manually onto a Microsoft Excel spreadsheet.

Patient and Public Involvement

No patients were involved nor was any patient data used in the development or analysis of this review.

Results

Literature Search Outcomes

Our search resulted in a total of 640 articles. The screening for eligibility is illustrated in Figure 1. A final total of 15 articles were included, with no additional articles identified from cross-referencing.

Confidential: For Review Only

Figure 1. PRISMA flowchart of identifying eligible studies.

Characteristics of Selected Studies

The studies included in this review focused on five main specialties: Cardiovascular surgery, General Surgery, (1 study shared with Urology), Neurosurgery, Respiratory Medicine and Gynaecology. Figure 2 breaks down the included studies by subspecialty.

The majority of eligible studies (n=10) assessed the use of 3DP models in CHD. Out of the 15 included studies, 3 assessed the utility of 3DP models amongst undergraduates (13–15). One study was a multi-centre study (10) and only 2 studies had followed-up participants after a period of time (13,16). The majority of studies were non-randomised, single-centred cross-sectional studies with no sample size calculations to determine the optimal number of study participants. Table 1 summarises the study design, participants and findings of the included studies.

Figure 2. Breakdown of specialties for included studies

Confidential: For Review Only

Study	Design and Objective	Study Cohort	Primary outcomes	Secondary outcomes
Assessing the utility of 3D Printed models of Mullerian tract anomalies for clinical education† (17)	Cohort study<br>Increased trainees' understanding of Mullerian tract anomalies and assess the feasibility of producing such models	Gynaecologists and general surgery trainees (exact number of participants not given)	3DP models were found to increase gynaecologists' understanding of Mullerian tract anomalies and their confidence in surgery to correct them.	None reported
Developing a 3D composite training model for cranial Remodelling (13)	Retrospective Cohort study<br>Use of 3DP models at 2 annual practical course to teach neurosurgical techniques (FAO and ES) for correction of craniosynostosis. Using realistic 3DP models were assessed as a tool for extraoperative training. Surveys were given to attendees and to non-attendees a year following the course.	33 Students, Residents, Fellows and Attendings over 2 years	Participants felt that the model was a valuable training tool for both surgical techniques and improved their understanding of and preparedness to perform the procedures.	None reported
Facilitating surgeon understanding of complex anatomy using a three-dimensional printed model (18)	Cross-sectional study<br>Use of 3DP to improve understanding of complex anatomy (conjoined twins) vs CT scan and Digital Reconstruction, in the context of pre-operative training/planning. Participants were allocated to different groups and were tested on anatomy identification, point-to-point distance and scale and shape of structures.	21 (12 Paediatric Surgery attendings, 9 Paediatric Surgery and General Surgery residents).	3DP models improved scale and shape orientation, identification of anatomy, but not linear/point-to-point distances.<br>Not all outcome data were numerically reported.<br>Participants using the 3DP were able to complete the examination significantly faster compared to CT alone and CT with digital reconstructions (6.6 min vs 18.9 min vs 14.9 min; p <0.05).	None reported
Hands-on surgical training of congenital heart surgery using 3-dimensional print models (10)	Cross-sectional study<br>Use of 3DP as part of surgical training for CHD. Participants were given a questionnaire following the surgical simulation session.	81 Cardiovascular surgery trainees and Attendings (experience 1-30 years), Sessions conducted in the USA, Canada and South Korea	3DP models were able to demonstrate the necessary pathological findings, and were deemed to be acceptable for surgical training. However, materials used for 3DP were different from real human myocardium.	Printing took 5-7 hours for each model at an estimated total cost of $150 to $210. The average cost for print materials per model was $60.
Incorporating Three-dimensional Printing into a Simulation-based Congenital Heart Disease and Critical Care Training Curriculum for Resident Physicians (19)	Cross-sectional study<br>Use of 3DP models in a 60-minute simulation teaching VSD anatomy, echocardiography, repair, and postoperative critical care management. Participants were given a pre- and post-session test scored out of 10.	23 Paediatric Resident Physicians	Participants report that the use of the 3DP models in the seminar improved their knowledge acquisition 4.83 vs 7.33 p=0.0082), knowledge reporting (4.25 vs 6.86 p=0.01) and structural conceptualisation (4.17 vs 7.22 p<0.0001).	None reported
Three-Dimensional Printed Pediatric Airway Model Improves Novice Learners'	Randomised control trial<br>To study the impact of 3DP airway models in training/teaching Flexible Bronchoscopy to	27 Paediatric residents (PGY2) at the beginning of their Paediatric	Intervention vs control groups: median post-study assessment score 6 vs 1.5 (p<0.001), median time taken 102 seconds vs 600 seconds (p<0.001), median difference in pre- and post-	Estimated total cost for materials and labour to trainer- $2,500. Cost of fusion

Confidential: For Review Only

Flexible Bronchoscopy Skills with Minimal Direct Teaching From Faculty (16)	paediatric residents with minimal experience, in terms of accuracy of landmark identification and time taken. Participants were assessed on identifying 6 structures on bronchoscopy and the time taken to complete the assessment. Control and intervention groups were assessed pre-study, post-study and at least 2 months following the session.	Respiratory rotation (18 in intervention group, 9 in control group).	study scores 4 vs 0 (p<0.001), median difference in times -432 seconds vs 0 seconds (p<0.001). At least 2 months post-study, intervention vs control: median post-study score 4 vs 2 (p=0.077), median time taken 240 seconds vs 576 seconds (p=0.036), median pre-study and delayed post-study score difference (2.5 vs 1 (p=0.123)), median difference in pre- and delayed post-study times -180 seconds vs 0 seconds (p=0.141).	deposition modeler-type 3D printer- $32,000.<br><br>12 hours to produce the computer model ready to print.
Three-dimensional printing models in congenital heart disease education for medical students: a controlled comparative study (14)	Randomised control trial<br>To compare knowledge acquisition and structural conceptualisation of 3 subtypes of VSD (perimembranous, subarterial and muscular) in medical students when 3DP models are utilised vs without 3DP models. Participants were given a post-session test assessing knowledge acquisition and structural conceptualisation, and subjective questionnaire.	63 Medical students (32 in intervention group, 31 in control)	3DP models significantly improved subjective understanding (mean score for intervention and control groups, out of 100: 72.19 vs 56.12 (p <0.0001)) and objective structural conceptualization (mean score for intervention and control groups, out of 30: 18.44 vs 14.52 (p=0.03)) but not in knowledge acquisition of VSD (mean score for intervention and control groups, out of 70: 44.06 vs 36.77 (p=0.06)).	None reported
Transcending Dimensions: a Comparative Analysis of Cloaca Imaging in Advancing the Surgeon's Understanding of Complex Anatomy (20)	Cross-sectional study<br>To compare the effectiveness of 4 different modalities in teaching cloacal malformations in the context of operative planning (2D contrast study cloacagram vs 3D rotatable CT scan reconstruction vs software-enhanced 3D video animation vs 3DP cloaca model).	59 paediatric surgeons (29 trainees and 30 attendings)	Participants allocated in 3DP sessions scored significantly better (p<0.001) compared to other modalities in both Trainee and Attending surgeon groups. 2D cloacogram vs 3D CT vs enhanced 3D CT vs 3DP; Trainees- 10.5% vs 67.1% vs 73.8%; Attendings- 22.2%, 54.8%, 66.2%, 74.0%.	18 hours to print. Cost of models not reported.
Usage of 3D models of tetralogy of Fallot for medical education: impact on learning congenital heart disease (21)	Randomised control trial<br>To compare conventional 2D drawings vs 3DP models in knowledge acquisition of tetralogy of Fallot. Pre- and post-session knowledge tests (out of 9) were performed, and self-reported questionnaires (out of 25) were completed.	35 Paediatric residents (17 in 2D image group, 18 in 3DP model group)	There was no observable significant difference between 3DP models and 2D images in terms of knowledge acquisition (mean post-test scores: 6.0 vs 6.3) or self-reported confidence in ToF (21 vs 20 (p=0.39)), but 3DP models provided significantly greater learner satisfaction (24 vs 21 (p=0.03)).	Cost to produce model ranges from $15 to $300 depending on the printer. Time taken to print each model was approximately 12 hours.
Use of 3D models of congenital heart disease as an education tool for cardiac nurses (22)	Cross-sectional study<br>Use of 3DP models to improve knowledge in cardiac nurses of various CHD after treatment: tetralogy of Fallot, transposition of the great arteries, aortic coarctation, pulmonary atresia, hypoplastic left heart syndrome. Participants were given a 5-question survey with Likert	100 cardiac nurses (65 paediatric, 35 adult)	60% agree/strongly agree that 3DP models improved their learning experience (improved understanding anatomy (86%), spatial orientation (70%), and anatomical complexity after treatment (66%)), 74% agree/strongly agree 3DP provided more information than diagrams. Participants found the models useful (mean score: 5.1 out of 7). The following qualitative themes were suggested by participants: the value	None reported

Confidential: For Review Only

	questions.		of being able to see inside the model heart explicitly, potential in education/training.	
Use of 3D models of vascular rings and slings to improve resident education (23)	Randomised control trial<br>Block randomisation was employed to recruit participants<br>Those in the intervention group used of 3DP models in didactic teaching sessions to improve understanding of Vascular rings and Pulmonary artery slings. The control group were only shown anterior, posterior and superior views of virtual models. Pre-session and post-session subjective questionnaires and knowledge testing were performed by participants.	36 Paediatric and Emergency Medicine Residents	Both groups self-report improved confidence in identifying, diagnosing and treating vascular rings and slings. Intervention groups scored significantly higher than the control groups (62.2% vs 45.1%; p=0.001), however the score improvement from pre-test to post-test scores were insignificant between intervention and control groups (2.6 vs 1.8; p=0.084).	None reported
Utility of three-dimensional models in resident education on simple and complex intracardiac congenital heart defects (24)	Randomised control trial<br>Block randomisation was employed to recruit participants<br>Both groups attended a lecture on either VSD or TOF with 2D images/virtual models, where the intervention group were given 3D printed models during the lecture as an adjunct to the lecture content. Pre-session and post-session subjective questionnaires and knowledge testing were performed by participants.	60 Paediatric and Emergency Medicine Residents (26 in the VSD portion of the study, and 34 in the TOF portion of the study)	Subjective reporting had significantly increased in both control and intervention groups in the VSD and TOF portions of the study. In the VSD study, the control group had a significantly greater improvement in post-test scores compared to those in the intervention arm (3.16 vs 1.93; p=0.004). In the TOF study, despite the intervention arm having a greater post-session test score than the control group (6.06 vs 5.29; p=0.037), there was no significant difference in pre-session and post-session score changes between control and intervention groups (2.23 vs 2.65; p=0.406).	None reported
Utility of 3D Printed Cardiac Models for Medical Student Education in Congenital Heart Disease: Across a Spectrum of Disease Severity (15)	Cross-sectional study<br>The impact of 3D printed models for PS, ASD, aortic coarctation, TOF, TGA, HLHS. The preferences for teaching methods were also explored. The study consisted of a 4-station workshop teaching participants the pathologies using 2D images, embryology videos, spoken explanation, pathology specimens and 3DP models. Students rotated between all stations over the course of the workshop. Participants were given a pre-station and post-station questionnaire at the 3D printed station assessing self-reported confidence on the conditions. At the end of the workshop, participants ranked the different teaching modalities on their utility and ranked	45 first year medical students	Using 3DP models improved self-reported confidence scores (PS (0.3, p< 0.001), ASD (0.6, p< 0.001), TOF (0.8, p< 0.001), d-TGA (0.9, p< 0.001), Coarct (0.8, p <0.001), HLHS (1.1, p< 0.001)). There was a strong correlation between the complexity of pathology and perceived knowledge increase (R^2 = 0.73, p=0.03). Participants agreed that they would use 3DP models in future teaching sessions (mean score: 4.40 out of 5). As teaching modalities 3DP models, pathology specimens, and spoken explanation were frequently ranked 3-5 out of 5 in utility (%of participants: 74.2% vs 80.7% vs 64.6%). Embryology videos and 2D diagrams were considered the least (% of participants scoring 1-2 out of 5: 58.0% vs 61.3%).	None reported

Confidential: For Review Only

	the utility of 3DP models for each pathology.			
Utility of 3-Dimensional Printing of Hearts with Complex Congenital Heart Disease in the Education of Pediatric Trainees and Sonographers† (25)	Cross-sectional study<br>Use of 3DP models vs CT/MRI imaging to improve general understanding of CHD	10 (5 Paediatric residents, 2 Sonographers and 2 Cardiology fellows)	80% of residents and 100% of sonographers and fellows felt that their understanding of CHD improved with 3DP models and felt that these should be utilised in the education of paediatric trainees	None reported
Utilizing Three-Dimensional Printing Technology to Assess the Feasibility of High-Fidelity Synthetic Ventricular Septal Defect Models for Simulation in Medical Education (26)	Cross-sectional study<br>Teaching and simulation using 3DP CHD models, including instruction on surgical incisions and suturing skills. Score out of 10	29 (16 Medical and 13 Pre-medical students)	Significant ($p<0.0001$) pre- and post-seminar score improvement in knowledge acquisition (3.22 vs 7.02), knowledge reporting (2.16 vs 6.60), and structural conceptualization (2.17 vs 6.31) of VSD	None reported

Table 1. Study characteristics of 3DP in Paediatric Medical Education. †Conference abstract. FOA: Fronto-orbital Advancement, ES: Endoscopic Suturectomy, VSD: ventricular septal defect, TOF: Tetralogy of Fallot, ASD: atrial septal defect, HLHS: hypoplastic left heart syndrome, TGA; transposition of the great arteries, PS: pulmonic stenosis, CHD: congenital heart disease.

Confidential: For Review Only

Primary outcomes

Studies reported a range of objective and subjective (self-reported) outcome measures across a range of parameters including knowledge acquisition, knowledge reporting, structural conceptualisation and identification, confidence in management and in explaining anatomy to others, learner satisfactions and increased subject interest. The key findings in each study are summarised in Table 1.

Objective assessment of 3DP models in the setting of CHDs had generally reported observable improvement in knowledge acquisition, structural conceptualisation and greater improvement from baseline assessment scores compared to control groups (19,24,26). However, observable differences were not always statistically significant (14,21,23). Those who used 3DP models self-reported improved subject understanding, user's confidence in reporting the pathology and in identifying, diagnosing and treating the pathology compared to controls (14,21,23,24). Participants felt that 3DP were more useful than diagrams and videos and would like for these models to be implemented in their usual training (15,22,25). However, from a surgical standpoint, whilst useful in training and demonstrating procedures, the textural properties of the material used did not match that of myocardial tissue (10).

The studies in paediatric gynaecology and general surgery revealed better self-reported and objective measured outcomes when using 3DP models (17,18,20). Identification of abdominal organs and cloacal malformations were more accurate and performed faster in those in the intervention arm of the studies compared to the control (18,20). 3DP models left participants with greater self-reported confidence in understanding Mullerian tract anomalies (17).

The two studies that had followed-up participants assessed the impact of 3DP on bronchoscopy and neurosurgical training (13,16). Those performing bronchoscopy on 3DP models compared to those

Confidential: For Review Only

who did not performed assessment tasks such as marker identification and completion time significantly better at immediate post-session testing. However these benefits were not seen after at least 2 months follow-up (16). Models produced for fronto-orbital advancement and endoscopic suturectomy simulation lead to improved understanding amongst attendees at the neurosurgical workshop, and at follow-up participants felt that they were more prepared compared to their colleagues who did not attend (13). This study, however, did not perform any quantitative analysis and did not objectively measure the impact 3DP models had at immediate or post-follow-up evaluation.

Cost and Duration of Model Generation

Four studies reported either the costs to or duration of generate 3DP models (10,16,20,21). Cost estimations varied widely, from $15 to $2500 depending on the type of printer, the material used, and whether other costs were included. Segmentation/model production time was also included for two of the CHD studies. This ranged from 5-7 hours per CHD model to print plus 1 hour for cleaning and dyeing (10); approximately 2 hours per TOF model segmentation and approximately 12 hours printing time (21). DeBoer et al claimed less than 12 hours for segmentation of an airway model (16). Gaisor reported that it took approximately 18 hours to print a 3D model demonstrating cloacal malformation (20).

Discussion

The 15 studies included in this review demonstrate objectively measured and user-reported benefit in using 3DP models in paediatric trainee and medical student training and educational settings in a range of subspecialties with a variety of outcomes. The quality of evidence, however, is diminished by a number of factors.

Confidential: For Review Only

Although no formal assessment of bias was performed, there are many limitations to the current evidence base. Many studies were single centred, cross-sectional studies with a small number of study participants and no long-term follow-up which produce lower quality results according to validated tools of assessing medical educational studies (27). The exception was one study which recruited participants from the USA, South Korea, and Canada (10).

A number of the studies did not take into account potential confounding factors. For instance, Smerling et al (15) measured the participants' self-reported understanding of CHDs immediately before and after exposure to 3DP CHD models. Each group entering the 3DP model station, however, had differing exposures depending on which group they were randomised to. Those who would have ended the workshop with the 3DP station would have watched an embryology video, attended a lecture on CHDs and examined pathology stations and may have reported greater confidence in understanding CHDs compared to those who started the workshop at the 3DP station.

The lack of randomisation, coupled with these studies not reporting how participants were recruited or randomised, is a source of selection bias. Some studies had failed to report baseline characteristics of participants (13,26) which means that the impact of using 3DP models may not be accurately reflected in outcome measurements. Cheng et al (13) had only analysed subjective outcome measures such as confidence in the pathology in focus, however objective measures were not scrutinised. Another source of bias in this study arises from the questions posed towards the control and intervention groups. Questions posed to the control group included “I understand the anatomy…” and “my training has fully prepared me for performing…”, whilst those in the intervention group were asked whether “…the model improved my understanding …” and “the model was a valuable training tool…”. Wording inconsistencies may lead to the questions becoming leading.

Confidential: For Review Only

In an educational setting, the reporting of knowledge retention and translatability of knowledge and skill retention is key. Despite this, only one study had reported long-term outcomes, which had shown that there was no significant difference after at least 2-months in terms of flexible bronchoscopy performance on a testing apparatus (16). No study had assessed if training procedures with 3DP had led to improved clinical outcomes. Outcome measures such as time taken to complete procedures in a clinical setting or procedural success/complication rates have been considered in studies focussing on simulation in medical education (28,29). It has also been demonstrated that using 3DP models for pre-operative planning have reduced operating theatre times by up to an hour (30). There is potential for future paediatric studies to evaluate the clinical translation of 3DP model utility in training.

Ten studies were based upon CHD, possibly due to CHDs being the most common birth defect (31). Traditional teaching of CHD has been delivered using 2D diagrams (for example, in textbooks), cross-sectional imaging modalities (MRI, CT, echocardiography), cadaveric specimens and off-the-shelf models (32). However, these tools pose a variety of problems, including difficulty in translating planar images into 3D reconstructions in the learner's mind and lack of tactile feedback in the case of imaging, and lack of availability of specific pathologies in the case of cadaveric specimens and plastic models. The subgroup of studies focussing on CHD have shown to overcome these issues, and have demonstrated increased understanding with more complex models (15). 3DP models have shown their strengths in simplifying complex concepts which has typically proven to be challenging with congenital defects (33) and therefore can be a great educational tool to implement into routine practice. This is especially useful for adult clinicians who will be involved in transition-to-adult-services to provide care for the growing population of adults with congenital diseases (34,35). As CHD proves to be difficult to those with minimal training (33) it would be beneficial for future studies assessing the use of 3DP models in non-paediatric clinicians.

Confidential: For Review Only

When considering the accuracy of models for procedure simulation or teaching, the model's size/shape and the suitability of the material used should be considered. The accuracy of model reconstruction has been demonstrated previously (36). In terms of material, acrylonitrile butadiene styrene was shown to mimic bone the most when used with a drill and craniotomy (13). However, replicating the consistency of soft tissue such as myocardium has been shown to be challenging (10). Whilst the motions of performing surgical techniques on a 1:1 scale model would be technically useful, the difference in material compliance would detract from the overall surgical experience. Another challenge would be the lack of surrounding structures and the inability to replicate bleeds or leaks, such as the of dural structures and cerebrospinal fluid (13). However, it has been demonstrated that soft tissues and artificial blood can be introduced to 3DP simulation models to replicate real patient anatomy and physiology (37). Whilst increasing the complexity of the models, improving accessibility and user-interface with 3D segmentation could ease their implementation in future paediatric simulation sessions.

Paediatric trainees in Low- and Middle-Income Countries (LMICs) face problems with lack of training opportunities due to lack of resources used traditionally to teach anatomy and pathophysiology (3,38). 3DP models may have a role in improving access to training resources in such countries. DeBoer and colleagues highlight their $2500 3DP training model costed 43-250% less than other commercially available trainers at time of publication, with simple maintenance requirements (16). 3DP models also confer the benefit of patient-specific pathologies that are seen in real-word clinical settings, as opposed to off-the-shelf models based upon generic anatomical structures which may not reflect real patient anatomy and pathological processes. Furthermore, the nature of the medium is highly conducive to collaboration. Using 3DP medical aids has shown benefit for patients in Sierra Leone (39), however there is a paucity of evidence in the use of the technology in paediatric teaching and training in LMICs, which would warrant further investigation into its cost-effectiveness.

Confidential: For Review Only

The current evidence base demonstrates a positive impact made by 3DP models on paediatric education and training objectively and subjectively for medical students and clinical trainees. The majority of studies failed to address sources of bias, assess clinically translatable outcomes, or evaluate the long-term benefits. Future studies should recruit larger cohorts, ideally at a multicentre level, randomise participants and adopt objective outcome measurements (e.g. anonymous anatomy examinations and monitoring of trainee clinical performance), including follow-up to assess long-term benefits. 3DP simulations also have the potential to be more sophisticated to imitate real scenarios with surrounding soft tissue structures and blood.

References

1. Royal College of Paediatrics and Child Health. RCPCH position paper in response to the Shape of Training Review. 2017 Oct.
2. Bindal T, Wall D, Goodyear HM. Medical students' views on selecting paediatrics as a career choice. Eur J Pediatr. 2011;170(9).
3. Sohail AH, Maan MHA, Sachal M, Soban M. Challenges of training and delivery of pediatric surgical services in developing economies: a perspective from Pakistan. BMC Pediatr. 2019 Dec 16;19(1).
4. Al-Yassin A, Long A, Sharma S, May J. How can general paediatric training be optimised in highly specialised tertiary settings? Twelve tips from an interview-based study of trainees. BMJ Paediatr Open. 2017 Sep 4;1(1).
5. Hilliard R, Bannister SL, Amin H, Baird B. Paediatric medical education: Challenges and new developments. Paediatr Child Health. 2009 May 1;14(5).
6. Regitz-Zagrosek V, Roos-Hesselink JW, Bauersachs J, Blomström-Lundqvist C, Cífková R, De Bonis M, et al. 2018 ESC Guidelines for the management of cardiovascular diseases during pregnancy. Eur Heart J. 2018 Sep 7;39(34).
7. Lichtenberger JP, Tatum PS, Gada S, Wyn M, Ho VB, Liacouras P. Using 3D Printing (Additive

Confidential: For Review Only

Manufacturing) to Produce Low-Cost Simulation Models for Medical Training. Mil Med [Internet]. 2018 Mar 1;183(suppl_1):73–7. Available from: https://academic.oup.com/milmed/article/183/suppl_1/73/4959929

8. Ganguli A, Pagan-Diaz GJ, Grant L, Cvetkovic C, Bramlet M, Vozenilek J, et al. 3D printing for preoperative planning and surgical training: a review. Biomed Microdevices [Internet]. 2018 Sep 4;20(3):65. Available from: http://link.springer.com/10.1007/s10544-018-0301-9

9. Giannopoulos AA, Mitsouras D, Yoo S-J, Liu PP, Chatzizisis YS, Rybicki FJ. Applications of 3D printing in cardiovascular diseases. Nat Rev Cardiol [Internet]. 2016 Dec 27;13(12):701–18. Available from: http://www.nature.com/articles/nrcardio.2016.170

10. Yoo SJ, Spray T, Austin EH, Yun TJ, van Arsdell GS. Hands-on surgical training of congenital heart surgery using 3-dimensional print models. J Thorac Cardiovasc Surg [Internet]. 2017;153(6):1530–40. Available from: http://dx.doi.org/10.1016/j.jtcvs.2016.12.054

11. Moher D, Liberati A, Tetzlaff J, Altman DG. Preferred Reporting Items for Systematic Reviews and Meta-Analyses: The PRISMA Statement. PLoS Med [Internet]. 2009 Jul 21;6(7):e1000097. Available from: https://dx.plos.org/10.1371/journal.pmed.1000097

12. Beller EM, Glasziou PP, Altman DG, Hopewell S, Bastian H, Chalmers I, et al. PRISMA for Abstracts: Reporting Systematic Reviews in Journal and Conference Abstracts. PLoS Med [Internet]. 2013 Apr 9;10(4):e1001419. Available from: https://dx.plos.org/10.1371/journal.pmed.1001419

13. Cheng D, Yuan M, Perera I, O'Connor A, Evins AI, Imahiyerobo T, et al. Developing a 3D composite training model for cranial remodeling. J Neurosurg Pediatr. 2019;24(6):632–41.

14. Su W, Xiao Y, He S, Huang P, Deng X. Three-dimensional printing models in congenital heart disease education for medical students: A controlled comparative study. BMC Med Educ. 2018;18(1):1–6.

15. Smerling J, Marboe CC, Lefkowitch JH, Pavlicova M, Bacha E, Einstein AJ, et al. Utility of 3D Printed Cardiac Models for Medical Student Education in Congenital Heart Disease: Across a

Confidential: For Review Only

Spectrum of Disease Severity. Pediatr Cardiol [Internet]. 2019;40(6):1258–65. Available from: https://doi.org/10.1007/s00246-019-02146-8

16. Deboer EM, Wagner J, Kroehl ME, Albietz J, Shandas R, Deterding RR, et al. Three-Dimensional Printed Pediatric Airway Model Improves Novice Learners' Flexible Bronchoscopy Skills with Minimal Direct Teaching from Faculty. Simul Healthc. 2018;13(4):284–8.

17. Hadden R, Grover S, Chuen J C-BJ. Assessing the Utility of 3D Printed Models of Mullerian Tract Anomalies for Clinical Education. Aust NEW Zeal J Obstet Gynaecol. 2018;58:55.

18. Cromeens BP, Ray WC, Hoehne B, Abayneh F, Adler B, Besner GE. Facilitating surgeon understanding of complex anatomy using a three-dimensional printed model. J Surg Res [Internet]. 2017;216:18–25. Available from: http://dx.doi.org/10.1016/j.jss.2017.04.003

19. Costello JP, Olivieri LJ, Su L, Krieger A, Alfares F, Thabit O, et al. Incorporating three-dimensional printing into a simulation-based congenital heart disease and critical care training curriculum for resident physicians. Congenit Heart Dis. 2015;10(2):185–90.

20. Gasior AC, Reck C, Lane V, Wood RJ, Patterson J, Strouse R, et al. Transcending Dimensions: a Comparative Analysis of Cloaca Imaging in Advancing the Surgeon's Understanding of Complex Anatomy. J Digit Imaging. 2019;32(5):761–5.

21. Loke YH, Harahsheh AS, Krieger A, Olivieri LJ. Usage of 3D models of tetralogy of Fallot for medical education: Impact on learning congenital heart disease. BMC Med Educ. 2017;17(1):1–8.

22. Biglino G, Capelli C, Koniordou D, Robertshaw D, Leaver LK, Schievano S, et al. Use of 3D models of congenital heart disease as an education tool for cardiac nurses. Congenit Heart Dis. 2017;12(1):113–8.

23. Jones TW, Seckeler MD. Use of 3D models of vascular rings and slings to improve resident education. Congenit Heart Dis. 2017;12(5):578–82.

24. White SC, Sedler J, Jones TW, Seckeler M. Utility of three-dimensional models in resident

Confidential: For Review Only

education on simple and complex intracardiac congenital heart defects. Congenit Heart Dis. 2018;13(6):1045–9.

25. D. P. Utility of 3 dimensional printing of hearts with complex congenital heart disease in the education of pediatric trainees and sonographers. FASEB J [Internet]. 2016;30(Meeting Abstracts):5–7. Available from: http://www.fasebj.org/content/30/1_Supplement/787.7.abstract?sid=a4993bad-734d-447f-a363-e524a7c6dee2%0Ahttp://ovidsp.ovid.com/ovidweb.cgi?T=JS&PAGE=reference&D=emed17&NEWS=N&AN=72317314

26. Costello JP, Olivieri LJ, Krieger A, Thabit O, Marshall MB, Yoo SJ, et al. Utilizing Three-Dimensional Printing Technology to Assess the Feasibility of High-Fidelity Synthetic Ventricular Septal Defect Models for Simulation in Medical Education. World J Pediatr Congenit Hear Surg. 2014;5(3):421–6.

27. Reed DA, Cook DA, Beckman TJ, Levine RB, Kern DE, Wright SM. Association Between Funding and Quality of Published Medical Education Research. JAMA [Internet]. 2007 Sep 5;298(9):1002. Available from: http://jama.jamanetwork.com/article.aspx?doi=10.1001/jama.298.9.1002

28. Zendejas B, Brydges R, Wang AT, Cook DA. Patient outcomes in simulation-based medical education: A systematic review. Journal of General Internal Medicine. 2013.

29. Griswold-Theodorson S, Ponnuru S, Dong C, Szyld D, Reed T, McGaghie WC. Beyond the Simulation Laboratory. Acad Med [Internet]. 2015 Nov;90(11):1553–60. Available from: http://journals.lww.com/00001888-201511000-00032

30. Tack P, Victor J, Gemmel P, Annemans L. 3D-printing techniques in a medical setting: a systematic literature review. Biomed Eng Online [Internet]. 2016 Dec 21;15(1):115. Available from: http://biomedical-engineering-online.biomedcentral.com/articles/10.1186/s12938-016-0236-4

Confidential: For Review Only

31. van der Linde D, Konings EEM, Slager MA, Witsenburg M, Helbing WA, Takkenberg JJM, et al. Birth Prevalence of Congenital Heart Disease Worldwide. J Am Coll Cardiol [Internet]. 2011 Nov;58(21):2241–7. Available from: https://linkinghub.elsevier.com/retrieve/pii/S0735109711030798

32. Seslar SP, Shepard CW, Giroud JM, Aiello VD, Cook AC, Spicer DE, et al. Lost treasures: a plea for the systematic preservation of cadaveric heart specimens through three-dimensional digital imaging. Cardiol Young [Internet]. 2015 Dec 16;25(8):1457–9. Available from: https://www.cambridge.org/core/product/identifier/S1047951115002589/type/journal_article

33. Webb CL, Jenkins KJ, Karpawich PP, Bolger AF, Donner RM, Allen HD, et al. Collaborative Care for Adults With Congenital Heart Disease. Circulation [Internet]. 2002 May 14;105(19):2318–23. Available from: https://www.ahajournals.org/doi/10.1161/01.CIR.0000017557.24261.A7

34. Viner RM. Transition of care from paediatric to adult services: one part of improved health services for adolescents: Figure 1. Arch Dis Child [Internet]. 2008 Feb;93(2):160–3. Available from: https://adc.bmj.com/lookup/doi/10.1136/adc.2006.103721

35. Marelli AJ, Mackie AS, Ionescu-Ittu R, Rahme E, Pilote L. Congenital Heart Disease in the General Population. Circulation [Internet]. 2007 Jan 16;115(2):163–72. Available from: https://www.ahajournals.org/doi/10.1161/CIRCULATIONAHA.106.627224

36. Schievano S, Migliavacca F, Coats L, Khambadkone S, Carminati M, Wilson N, et al. Percutaneous Pulmonary Valve Implantation Based on Rapid Prototyping of Right Ventricular Outflow Tract and Pulmonary Trunk from MR Data. Radiology [Internet]. 2007 Feb;242(2):490–7. Available from: http://pubs.rsna.org/doi/10.1148/radiol.2422051994

37. O'Reilly MK, Reese S, Herlihy T, Geoghegan T, Cantwell CP, Feeney RNM, et al. Fabrication and assessment of 3D printed anatomical models of the lower limb for anatomical teaching and femoral vessel access training in medicine. Anat Sci Educ [Internet]. 2016 Jan;9(1):71–9. Available from: http://doi.wiley.com/10.1002/ase.1538

Confidential: For Review Only

38. Cass H, Barclay S, Gerada C, Lumsden DE, Sritharan K. Complexity and challenge in paediatrics: a roadmap for supporting clinical staff and families. Arch Dis Child [Internet]. 2019 Jun 11;archdischild-2018-315818. Available from: https://adc.bmj.com/lookup/doi/10.1136/archdischild-2018-315818

39. van der Stelt M, Verhulst AC, Vas Nunes JH, Koroma TAR, Nolet WWE, Slump CH, et al. Improving Lives in Three Dimensions: The Feasibility of 3D Printing for Creating Personalized Medical Aids in a Rural Area of Sierra Leone. Am J Trop Med Hyg [Internet]. 2020 Apr 1;102(4):905–9. Available from: http://www.ajtmh.org/content/journals/10.4269/ajtmh.19-0359

Funding statement

The authors received no specific grant from any funding agency in the public, commercial or not-for-profit sectors for conducting this review. The authors acknowledge the generous support of the British Heart Foundation (CH/17/1/32804) and The Grand Appeal (Bristol Children's Hospital Charity).

Competing interests statement

The authors declare that the research was conducted in the absence of any commercial or financial relationships that could be construed as a potential conflict of interest.

Patient consent for publication

Not required.

Ethics approval

Confidential: For Review Only

Ethical approval was not required as no primary patient data was used in this review.

Data availability statement

All data relevant to the study are included in the article or uploaded as supplemental information.

No additional information.

Author contributions

Ashar Asif conceived the study and drafted the study protocol, registered the review to Prospero, conducted the review searches, was first reviewer of the eligible studies and lead the writing of the manuscript.

Elgin Meng Lee reviewed eligible papers and contributed to the writing of the manuscript.

Massimo Caputo assisted in editing the final manuscript.

Giovanni Biglino assisted in editing the final manuscript.

Andrew Shearn conceived and supervised the study, was adjudicator for any conflicts in study selection for the review, and assisted in editing the final manuscript.

All authors have read and approvedf the final manuscript.

Confidential: For Review Only

Section/topic	#	Checklist item	Reported on page #
**TITLE**			
Title	1	Identify the report as a systematic review, meta-analysis, or both.	1
**ABSTRACT**			
Structured summary	2	Provide a structured summary including, as applicable: background; objectives; data sources; study eligibility criteria, participants, and interventions; study appraisal and synthesis methods; results; limitations; conclusions and implications of key findings; systematic review registration number.	2-3
**INTRODUCTION**			
Rationale	3	Describe the rationale for the review in the context of what is already known.	4
Objectives	4	Provide an explicit statement of questions being addressed with reference to participants, interventions, comparisons, outcomes, and study design (PICOS).	4
**METHODS**			
Protocol and registration	5	Indicate if a review protocol exists, if and where it can be accessed (e.g., Web address), and, if available, provide registration information including registration number.	5
Eligibility criteria	6	Specify study characteristics (e.g., PICOS, length of follow-up) and report characteristics (e.g., years considered, language, publication status) used as criteria for eligibility, giving rationale.	6
Information sources	7	Describe all information sources (e.g., databases with dates of coverage, contact with study authors to identify additional studies) in the search and date last searched.	5
Search	8	Present full electronic search strategy for at least one database, including any limits used, such that it could be repeated.	5 (on Prospero registration)
Study selection	9	State the process for selecting studies (i.e., screening, eligibility, included in systematic review, and, if applicable, included in the meta-analysis).	5
Data collection process	10	Describe method of data extraction from reports (e.g., piloted forms, independently, in duplicate) and any processes for obtaining and confirming data from investigators.	6
Data items	11	List and define all variables for which data were sought (e.g., PICOS, funding sources) and any assumptions and simplifications made.	5

Confidential: For Review Only

Risk of bias in individual studies	12	Describe methods used for assessing risk of bias of individual studies (including specification of whether this was done at the study or outcome level), and how this information is to be used in any data synthesis.	N/A
Summary measures	13	State the principal summary measures (e.g., risk ratio, difference in means).	N/A
Synthesis of results	14	Describe the methods of handling data and combining results of studies, if done, including measures of consistency (e.g., I^2) for each meta-analysis.	N/A
Risk of bias across studies	15	Specify any assessment of risk of bias that may affect the cumulative evidence (e.g., publication bias, selective reporting within studies).	N/A
Additional analyses	16	Describe methods of additional analyses (e.g., sensitivity or subgroup analyses, meta-regression), if done, indicating which were pre-specified.	N/A
**RESULTS**			
Study selection	17	Give numbers of studies screened, assessed for eligibility, and included in the review, with reasons for exclusions at each stage, ideally with a flow diagram.	7
Study characteristics	18	For each study, present characteristics for which data were extracted (e.g., study size, PICOS, follow-up period) and provide the citations.	7-13
Risk of bias within studies	19	Present data on risk of bias of each study and, if available, any outcome level assessment (see item 12).	N/A
Results of individual studies	20	For all outcomes considered (benefits or harms), present, for each study: (a) simple summary data for each intervention group (b) effect estimates and confidence intervals, ideally with a forest plot.	9-13
Synthesis of results	21	Present results of each meta-analysis done, including confidence intervals and measures of consistency.	N/A
Risk of bias across studies	22	Present results of any assessment of risk of bias across studies (see Item 15).	N/A
Additional analysis	23	Give results of additional analyses, if done (e.g., sensitivity or subgroup analyses, meta-regression [see Item 16]).	N/A
**DISCUSSION**			
Summary of evidence	24	Summarize the main findings including the strength of evidence for each main outcome; consider their relevance to key groups (e.g., healthcare providers, users, and policy makers).	15-18

Confidential: For Review Only

Limitations	25	Discuss limitations at study and outcome level (e.g., risk of bias), and at review-level (e.g., incomplete retrieval of identified research, reporting bias).	16-18
Conclusions	26	Provide a general interpretation of the results in the context of other evidence, and implications for future research.	18
**FUNDING**			
Funding	27	Describe sources of funding for the systematic review and other support (e.g., supply of data); role of funders for the systematic review.	24

481

Confidential: For Review Only

BMJ Paediatrics Open

The Role of 3D Printing Technology in Paediatric Teaching and Training: a Systematic Review

Journal:	*BMJ Paediatrics Open*
Manuscript ID	bmjpo-2021-001050.R1
Article Type:	Original research
Date Submitted by the Author:	07-Apr-2021
Complete List of Authors:	Asif, Ashar; University of Bristol Faculty of Medicine and Dentistry,<br>Lee, Elgin; Newcastle Upon Tyne Hospitals NHS Foundation Trust,<br>Caputo, Massimo; University of Bristol Faculty of Medicine and Dentistry; University of Bristol School of Clinical Science, Bristol Heart Institute<br>Biglino, Giovanni; University of Bristol School of Clinical Science, Bristol Heart Institute; Imperial College London National Heart and Lung Institute<br>Shearn, Andrew; University of Bristol Faculty of Medicine and Dentistry; University of Bristol School of Clinical Science, Bristol Heart Institute
Keywords:	Data Collection, Information Technology, Technology, Cardiology, Neurosurgery

SCHOLARONE™
Manuscripts

I, the Submitting Author has the right to grant and does grant on behalf of all authors of the Work (as defined in the below author licence), an exclusive licence and/or a non-exclusive licence for contributions from authors who are: i) UK Crown employees; ii) where BMJ has agreed a CC-BY licence shall apply, and/or iii) in accordance with the terms applicable for US Federal Government officers or employees acting as part of their official duties; on a worldwide, perpetual, irrevocable, royalty-free basis to BMJ Publishing Group Ltd ("BMJ") its licensees and where the relevant Journal is co-owned by BMJ to the co-owners of the Journal, to publish the Work in this journal and any other BMJ products and to exploit all rights, as set out in our licence.

The Submitting Author accepts and understands that any supply made under these terms is made by BMJ to the Submitting Author unless you are acting as an employee on behalf of your employer or a postgraduate student of an affiliated institution which is paying any applicable article publishing charge ("APC") for Open Access articles. Where the Submitting Author wishes to make the Work available on an Open Access basis (and intends to pay the relevant APC), the terms of reuse of such Open Access shall be governed by a Creative Commons licence – details of these licences and which Creative Commons licence will apply to this Work are set out in our licence referred to above.

Other than as permitted in any relevant BMJ Author's Self Archiving Policies, I confirm this Work has not been accepted for publication elsewhere, is not being considered for publication elsewhere and does not duplicate material already published. I confirm all authors consent to publication of this Work and authorise the granting of this licence.

Confidential: For Review Only

The Role of 3D Printing Technology in Paediatric Teaching and Training: a Systematic Review

Confidential: For Review Only

Ashar Asif[1], ORCiD 0000-0002-9361-1083

Elgin Meng Lee[2] ORCiD 0000-0002-0054-1574

Massimo Caputo[1, 3] ORCiD 0000-0001-7508-0891

Giovanni Biglino[3, 4] ORCiD 0000-0003-0413-149X

Andrew I. U. Shearn[1, 3] ORCiD 0000-0002-3741-9118

1. Bristol Medical School, University of Bristol, Bristol Royal Infirmary, Bristol, UK, BS2 8HW,

2. Newcastle upon Tyne Hospitals NHS Foundation Trust, Freeman Road, Newcastle upon Tyne, UK, NE7 7DN

3. Bristol Heart Institute, Bristol Royal Infirmary, Bristol, UK, BS2 8HW

4. National Heart and Lung Institute, Guy Scadding Building, Imperial College London, London, UK, SW3 6LY

Corresponding author

Dr Andrew Shearn

University of Bristol

Research Floor Level 7

Bristol Royal Infirmary

Upper Maudlin Street

Bristol BS2 8HW

Tel: (44) 0117 3423142

Email: andrew.shearn@bristol.ac.uk

Word count: 2499

Abstract

Background

In the United Kingdom, undergraduate paediatric training is brief resulting in trainees with a lower paediatric knowledge base compared to other aspects of medicine. With congenital conditions being successfully treated at childhood, adult clinicians encounter and will need to understand these complex pathologies. Patient-specific 3D printed (3DP) models have been used in clinical training, especially for rarer, complex conditions. We perform a systematic review to evaluate the evidence base in using 3DP models to train paediatricians, surgeons, medical students, and nurses.

Methods

Online databases PubMed, Web of Science, EMBASE and were searched between January 2010 and April 2020 using search terms relevant to “paediatrics”, “education”, “training” and “3D printing”. Participants were medical students, postgraduate trainees or clinical staff. Comparative studies (patient-specific 3DP models vs. traditional teaching methods) and non-comparative studies were included. Outcomes gauged objective and subjective measures; test scores, time taken to complete tasks, self-reported confidence and personal preferences on 3DP models. If reported, the cost of, and time taken, to produce the models were noted.

Confidential: For Review Only

Results

From 587 results, 15 studies fit the criteria of the review protocol, with 5/15 being randomised-control studies and 10/15 focussing on cardiovascular conditions. Participants using 3DP models had greater improvement in test scores and faster times to complete procedures and identify anatomical landmarks compared to traditional teaching methods (2D diagrams, lectures, videos, supervised clinical events). User feedback was positive, reporting greater user self-confidence in understanding concepts with users wishing for integrated use of 3DP in regular teaching. Four studies reported the costs and times of production, which varied depending on model complexity and printer. 3DP models were cheaper than “off-the-shelf” models available on the market and with the benefit of utilising real-world pathologies. These mostly non-randomised and single-centred studies did not address bias or report long-term or clinically translatable outcomes.

Conclusions

3DP models were associated with greater user satisfaction and good short-term educational outcomes, with low quality evidence. Multicentred, randomised studies with long-term follow-up and clinically assessed outcomes are needed to fully assess the benefits in this setting.

Systematic review registration

The review is registered on Prospero. Prospero ID: CRD42020179656

Available from: https://www.crd.york.ac.uk/prospero/display_record.php?ID=CRD42020179656

Confidential: For Review Only

What is known about the subject

- Undergraduate paediatric education is known to be short and postgraduate paediatric training can be limited within certain regions.
- Patients with congenital diseases are surviving to adulthood, meaning adult clinicians are seeing an increase of cases which were traditionally considered as 'paediatric conditions' .
- Patient specific 3D printed models have been used in other medical and surgical specialties for hands-on education and training and can compensate for these pitfalls.

What this review adds

- 3D printed models in paediatric education and training sessions have been shown to improve immediate educational and procedural performance, with high user satisfaction.
- The quality of evidence is poor due to unaddressed confounding factors, small study cohorts and poor study design.
- In the few studies that reported costs, 3D printed models had the benefit of producing models at relatively lower costs compared to alternative resources.

Introduction

In 2017, the Royal College of Paediatrics and Child Health published a response to the "Shape of Training Review" of postgraduate training within the United Kingdom. They observed that "doctors coming into paediatric specialty training do so from a lower knowledge base than adult medicine" (1). This could partly be explained by the brevity of undergraduate exposure to paediatrics, typically 4-8 weeks during the entirety of medical school (2). Furthermore some paediatric trainees report a lack of educational opportunities within their postgraduate training programme (3), potentially driven by the varied facilities offered by different regions and centres (4,5).

Confidential: For Review Only

It is increasingly important for non-paediatric clinicians to fully appreciate paediatric pathologies. Novel therapies have allowed many children, who would have had poor outcomes from previously life-limiting diseases, to now live with such pathologies resulting in complex patients with chronic conditions (5). Clinical care and observation over the adult lifetime of these patients is often overseen by a myriad of clinicians, frequently requiring specialised oversight such as in the case of pregnant women with congenital heart disease (CHD) (6).

In recent years, 3D printing (3DP) has played a role in a variety of medical and surgical training and educational settings (7–9). 3DP models can replicate in-vivo clinical pathologies based on radiological images, allowing trainees to be exposed to pathologies and potential procedures they would not ordinarily experience through their routine clinical exposure (10). We aim to evaluate the current evidence base regarding the utility of 3DP compared to traditional teaching methods (i.e. 2D images/diagrams, lectures, videos, ward-based teaching) in educating and training of medical students and clinical trainees in paediatrics.

Methods

Study Protocol

The study protocol is registered on PROSPERO and can be found with the following ID: CRD42020179656. Throughout the review, the authors referred and adhered to the Preferred Reporting Items for Systematic reviews and Meta-Analyses (PRISMA) guidelines (11).

Literature search strategy

Confidential: For Review Only

The databases PubMed, Web of Science, EMBASE and were searched. The search included all published material since January 2010 (last accessed in April 2020) in the English language. This timeframe was selected to ensure the studies reviewed used the most recent available technology. The following terms were used across all databases: ((3d-prin*) OR (3d prin*) OR (three-dimensional print*) OR (three dimensional print*) OR (3-d print*)) AND ((paediat*) OR (pediat*)) AND ((education) OR (teaching) OR (training) OR (session) OR (simulation)). Synonyms and spelling variations were also searched to allow for international spellings and alternative terms (e.g. paediatrics vs. pediatrics). Searches on PubMed included MeSH headings.

Selection criteria

The inclusion criteria for final review were studies using 3DP models derived from paediatric patient imaging data (e.g. computed tomography (CT), magnetic resonance imaging (MRI), ultrasound (US) or echocardiography) and that occurred in an educational or training setting. This was defined as a pre-planned session or programme as part of an educational syllabus, training programme or professional course separate from a clinical setting and not aimed to directly contribute towards patient outcomes. This excluded studies which used 3DP models to facilitate communication with patients, carers or clinical staff. Studies that produced a 3DP model for pre-operative planning for a specific case were also excluded. Included studies evaluated the use of patient-specific models with physicians, surgeons, nurses and allied health professionals undergoing teaching, training or simulation.. Comparative studies would compare the use of 3DP models against traditional educational methods such as lectures, tutorials, practical sessions, or textbooks. Case reports and case series were also included if they used the models in an educational setting and reported educational outcome measures. Published conference abstracts were included as per PRISMA guidance to minimise publication bias (12). Primary outcome measures were either objective measures such as pre-/post-intervention testing scores or procedure performance, or subjective

Confidential: For Review Only

measures such as participant reported opinions from questionnaires. Additional outcome measures that were recorded included the time taken to produce models and the cost of model production.

Data extraction and appraisal of evidence

The titles and abstracts of all papers obtained during the searches were screened for relevance by two authors independently (AA, EL). Relevant papers were then independently assessed for eligibility in full against the study protocol (AA, EL). Any disagreements were resolved by consensus, with the senior author (AS) acting as a final adjudicator for any unresolved discrepancies. The final included papers were then reviewed and appraised by AA and EL. Data was extracted manually onto a Microsoft Excel spreadsheet.

Patient and Public Involvement

No patients were involved nor was any patient data used in the development or analysis of this review.

Results

Literature Search Outcomes

Our search resulted in a total of 587 articles. The screening for eligibility is illustrated in Figure 1. A final total of 15 articles were included, with no additional articles identified from cross-referencing.

Figure 1. PRISMA flowchart of identifying eligible studies.

Confidential: For Review Only

Characteristics of Selected Studies

The studies included in this review focused on five main specialties relating to paediatric or congenital disorders: Cardiovascular surgery, General Surgery, (1 study shared with Urology), Neurosurgery, Respiratory Medicine and Gynaecology. Figure 2 breaks down the included studies by subspecialty.

The majority of eligible studies (n=10) assessed the use of 3DP models in CHD. Out of the 15 included studies, 3 assessed the utility of 3DP models amongst undergraduates (13–15). One study was a multi-centre study (10) and only 2 studies had followed-up participants after a period of time (13,16). The majority of studies were non-randomised, single-centred cross-sectional studies with no sample size calculations to determine the optimal number of study participants. Tables 1, 2 and summarise the study design, participants and findings of the included studies.

Figure 2. Breakdown of specialties for included studies

Confidential: For Review Only

Study	***Design and Objective***	***Study Cohort***	***Primary outcomes***	***Secondary outcomes***
Facilitating surgeon understanding of complex anatomy using a three-dimensional printed model (17)	Use of 3DP to improve understanding of complex anatomy (conjoined twins) vs CT scan and Digital Reconstruction	21 (12 Paediatric Surgery Attendings, 9 Paediatric Surgery and General Surgery Residents).	3DP models improved scale and shape orientation, identification of anatomy, but not linear/point-to-point distances.<br><br>Not all outcome data were reported.<br><br>Using 3DP models was associated improved test times compared to CT alone and CT with digital reconstructions (6.6 min vs 18.9 min vs 14.9 min; p <0.05).	None reported
Hands-on surgical training of congenital heart surgery using 3-dimensional print models (10)	Use of 3DP as part of surgical training for CHD. Participants were given a questionnaire following the surgical simulation session.	81 Cardiovascular surgery trainees and Attendings. Conducted in the USA, Canada and South Korea	3DP models demonstrated necessary pathological findings, and were acceptable for surgical training. Materials used differed from real human myocardium.	Printing took 5-7 hours for each model at an estimated total cost of $150 to $210. The average cost for print materials per model was $60.
Incorporating Three-dimensional Printing into a Simulation-based Congenital Heart Disease and Critical Care Training Curriculum for Resident Physicians (18)	Use of 3DP models in a 60-minute simulation teaching VSD anatomy, echocardiography, repair, and postoperative critical care management. Participants were given a pre- and post-session test scored out of 10.	23 Paediatric Resident Physicians	3DP models improved their knowledge acquisition 4.83 vs 7.33 p=0.0082), knowledge reporting (4.25 vs 6.86 p=0.01) and structural conceptualisation (4.17 vs 7.22 p<0.0001).	None reported
Transcending Dimensions: a Comparative Analysis of Cloaca Imaging in Advancing the Surgeon's Understanding of Complex Anatomy (19)	To compare the effectiveness of 4 different modalities in teaching cloacal malformations in the context of operative planning (2D contrast study cloacagram vs 3D rotatable CT scan reconstruction vs software-enhanced 3D video animation vs 3DP cloaca model).	59 paediatric surgeons (29 trainees and 30 Attendings)	Use of 3DP models was associated with significantly better (p<0.001) compared to other modalities. 2D cloacogram vs 3D CT vs enhanced 3D CT vs 3DP; Trainees- 10.5% vs 67.1% vs 73.8%; Attendings- 22.2%, 54.8%, 66.2%, 74.0%.	18 hours to print. Cost of models not reported.
Use of 3D models of congenital heart disease as an education tool for cardiac nurses (20)	Use of 3DP models to improve knowledge in cardiac nurses of various CHD after treatment: tetralogy of Fallot, transposition of the great arteries, aortic coarctation, pulmonary atresia, hypoplastic left heart syndrome. Participants were given a 5-question survey with Likert questions.	100 cardiac nurses (65 paediatric, 35 adult)	Percentage of participants that agree/strongly agree: 3DP models:<br>• improved their learning experience: 60%,<br>• improved understanding anatomy: 86%<br>• spatial orientation: 70%<br>• anatomical complexity after treatment: 66%,<br>• provided more information than diagrams: 74%.	None reported

Confidential: For Review Only

			Mean score for utility: 5.1 out of 7.<br><br>Participants reported the value of being able to see inside the model heart explicitly and potential of using 3DP models in education/training.	
Utility of 3D Printed Cardiac Models for Medical Student Education in Congenital Heart Disease: Across a Spectrum of Disease Severity (15)	The impact of 3D printed models for PS, ASD, aortic coarctation, TOF, TGA, HLHS. A 4-station workshop used 2D images, embryology videos, spoken explanation, pathology specimens and 3DP models. Students rotated between all stationsand were given a pre-station and post-station questionnaire at the 3D printed station assessing self-reported confidence and ranked the different teaching modalities.	45 first year medical students	Using 3DP models improved self-reported confidence scores (PS (0.3, p< 0.001), ASD (0.6, p< 0.001), TOF (0.8, p< 0.001), d-TGA (0.9, p< 0.001), Coarct (0.8, p <0.001), HLHS (1.1, p< 0.001)).<br><br>Strong correlation between the complexity of pathology and perceived knowledge increase (R^2 = 0.73, p=0.03).<br><br>Participants agreed that they would use 3DP models in future teaching sessions (mean score: 4.40 out of 5).<br><br>74.2% of participants scored 3DP models between 3-5 out of 5 in utility..	None reported
Utility of 3-Dimensional Printing of Hearts with Complex Congenital Heart Disease in the Education of Pediatric Trainees and Sonographers† (21)	Use of 3DP models vs CT/MRI imaging to improve general understanding of CHD	10 (5 Paediatric Residents, 2 Sonographers and 2 Cardiology Fellows)	80% of residents and 100% of sonographers and fellows felt that their understanding of CHD improved with 3DP models and felt that these should be utilised in the education of paediatric trainees	None reported
Utilizing Three-Dimensional Printing Technology to Assess the Feasibility of High-Fidelity Synthetic Ventricular Septal Defect Models for Simulation in Medical Education (22)	Teaching and simulation using 3DP CHD models, including instruction on surgical incisions and suturing skills. Score out of 10	29 (16 Medical and 13 Pre-medical students)	Significant (p<0.0001) pre- and post-seminar score improvement in knowledge acquisition (3.22 vs 7.02), knowledge reporting (2.16 vs 6.60), and structural conceptualization (2.17 vs 6.31) of VSD	None reported

Table 1. Study characteristics of cross-sectional studies. †Conference abstract. VSD: ventricular septal defect, TOF: Tetralogy of Fallot, ASD: atrial septal defect, HLHS: hypoplastic left heart syndrome, TGA; transposition of the great arteries, PS: pulmonic stenosis, CHD: congenital heart disease.

Confidential: For Review Only

Study	***Design and Objective***	***Study Cohort***	***Primary outcomes***	***Secondary outcomes***
Assessing the utility of 3D Printed models of Mullerian tract anomalies for clinical education† (23)	Impact of 3DP models on trainees' understanding of Mullerian tract anomalies and assess the feasibility of producing such models	Gynaecologists and general surgery trainees (exact number of participants not given)	3DP models were found to increase gynaecologists' understanding of Mullerian tract anomalies and their confidence in surgery to correct them.	None reported
Developing a 3D composite training model for cranial Remodelling (13)	Use of 3DP models at 2 annual practical course to teach neurosurgical techniques (FAO and ES) for correction of craniosynostosis. Surveys were given to attendees and non-attendees a year following the course.	33 Students, Resident, Fellow and Attending surgeons over 2 years	Model were a valuable training tool for surgical techniques and improved understanding and preparedness to perform the procedures.	None reported
Study	***Design and Objective***	***Study Cohort***	***Primary outcomes***	***Secondary outcomes***
Three-Dimensional Printed Pediatric Airway Model Improves Novice Learners' Flexible Bronchoscopy Skills with Minimal Direct Teaching From Faculty (16)	The impact of 3DP airway models in training/teaching Flexible Bronchoscopy to paediatric residents. Assessed on identifying 6 structures on bronchoscopy and the time taken to complete. Control and intervention groups were assessed pre-study, post-study and at least 2 months following the session.	27 Paediatric Residents (PGY2) at the beginning of their Paediatric Respiratory rotation (18 in intervention group, 9 in control group).	Intervention vs control groups: median difference in pre- and post-study scores 4 vs 0 ($p<0.001$), median difference in times -432 seconds vs 0 seconds ($p<0.001$).<br><br>At least 2 months post-study, intervention vs control: median pre-study and delayed post-study score difference (2.5 vs 1 ($p=0.123$)), median difference in pre- and delayed post-study times -180 seconds vs 0 seconds ($p=0.141$).	Estimated total cost for materials and labour to trainer- $2,500. Cost of fusion deposition modeler-type 3D printer- $32,000.<br><br>12 hours to produce the computer model ready to print.
Three-dimensional printing models in congenital heart disease education for medical students: a	To compare knowledge acquisition and structural conceptualisation of 3 subtypes of VSD in medical students with 3DP models vs without 3DP models. Participants were given a	63 Medical students (32 in intervention group, 31 in control)	3DP models significantly improved subjective understanding (mean score for intervention and control groups, out of 100: 72.19 vs 56.12 ($p<0.0001$)) and objective structural conceptualization (mean score for intervention and control	None reported

Table 2. Study characteristics of cohort studies. †Conference abstract. FOA: Fronto-orbital Advancement, ES: Endoscopic Suturectomy.

Confidential: For Review Only

controlled comparative study (14)	post-session test assessing knowledge acquisition and structural conceptualisation, and subjective questionnaire.		groups, out of 30: 18.44 vs 14.52 (p=0.03)) but not in knowledge acquisition of VSD (mean score for intervention and control groups, out of 70: 44.06 vs 36.77 (p=0.06)).	
Usage of 3D models of tetralogy of Fallot for medical education: impact on learning congenital heart disease (24)	To compare conventional 2D drawings vs 3DP models in knowledge acquisition of TOF. Pre- and post-session knowledge tests (out of 9) were performed, and self-reported questionnaires (out of 25) were completed.	35 Paediatric Residents (17 in 2D image group, 18 in 3DP model group)	No observable significant difference between 3DP models and 2D images in terms of knowledge acquisition (mean post-test scores: 6.0 vs 6.3) or self-reported confidence in ToF (21 vs 20 (p=0.39)), but 3DP models provided significantly greater learner satisfaction (24 vs 21 (p=0.03)).	Cost to produce model ranges from $15 to $300 depending on the printer. Time taken to print each model was approximately 12 hours.
Use of 3D models of vascular rings and slings to improve resident education (25)	Block randomisation was employed to recruit participants<br><br>The intervention group used of 3DP models in didactic teaching sessions to improve understanding of Vascular rings and Pulmonary artery slings. The control group were shown virtual models. Pre-session and post-session subjective questionnaires and knowledge testing were performed by participants.	36 Paediatric and Emergency Medicine Residents	Both groups self-report improved confidence in identifying, diagnosing and treating vascular rings and slings. Intervention groups scored significantly higher than the control groups (62.2% vs 45.1%; p=0.001), however the score improvement from pre-test to post-test scores were insignificant between intervention and control groups (2.6 vs 1.8; p=0.084).	None reported
Utility of three-dimensional models in resident education on simple and complex intracardiac congenital heart defects (26)	Block randomisation was employed to recruit participants<br><br>Both groups attended a lecture with 2D images/virtual models. The intervention group were given 3D printed models during the lecture as an adjunct to the lecture content. Pre-session and post-session subjective questionnaires and knowledge testing were performed by participants.	60 Paediatric and Emergency Medicine Residents (26 in the VSD portion of the study, and 34 in the TOF portion of the study)	Subjective reporting had significantly increased in both control and intervention groups in the VSD and TOF portions of the study. In the VSD study, the control group had a significantly greater improvement in post-test scores compared to those in the intervention arm (3.16 vs 1.93; p=0.004). In the TOF study, despite the intervention arm having a greater post-session test score than the control group (6.06 vs 5.29; p=0.037), there was no significant difference in pre-session and post-session score changes between control and intervention groups (2.23 vs 2.65; p=0.406).	None reported

Table 3. Study characteristics of randomised control trials. VSD: ventricular septal defect, TOF: Tetralogy of Fallot.

Confidential: For Review Only

Primary outcomes

Studies reported a range of objective and subjective (self-reported) outcome measures across a range of parameters including knowledge acquisition, knowledge reporting, structural conceptualisation and identification, confidence in management and in explaining anatomy to others, learner satisfactions and increased subject interest. The key findings in each study are summarised in Table 1.

Objective assessment of 3DP models in the setting of CHDs had generally reported observable improvement in knowledge acquisition, structural conceptualisation and greater improvement from baseline assessment scores compared to control groups (18,22,26). However, observable differences were not always statistically significant (14,24,25). Those who used 3DP models reported improved subject understanding, user confidence in reporting the pathology and in identifying, the pathology compared to controls (14,24–26). Participants felt that 3DP were more useful than diagrams and videos and would like for these models to be implemented in their usual training (15,20,21). However, from a surgical standpoint, whilst useful in training and demonstrating procedures, the textural properties of the material used did not match that of myocardial tissue (10).

The studies in paediatric gynaecology and general surgery revealed better self-reported and objective measured outcomes when using 3DP models (17,19,23). Identification of abdominal organs and cloacal malformations were more accurate and performed faster in those in the intervention arm of the studies compared to the control (17,19). 3DP models left participants with greater self-reported confidence in understanding Mullerian tract anomalies (23).

Confidential: For Review Only

The two studies that followed-up participants assessed the impact of 3DP on bronchoscopy and neurosurgical training (13,16). Those performing bronchoscopy on 3DP models compared to those who did not performed assessment tasks such as marker identification and completion time significantly better at immediate post-session testing. However these benefits were not seen after at least 2 months follow-up (16). Models produced for fronto-orbital advancement and endoscopic suturectomy simulation lead to improved understanding amongst attendees at the neurosurgical workshop, and at follow-up participants felt that they were more prepared compared to their colleagues who did not attend (13). This study, however, did not perform any quantitative analysis and did not objectively measure the impact 3DP models had at immediate or post-follow-up evaluation.

Cost and Duration of Model Generation

Four studies reported either the costs to or duration of generate 3DP models (10,16,19,24). Cost estimations varied widely, from $15 to $2500 depending on the type of printer, the material used, and whether other costs were included. Segmentation/model production time was also included for two of the CHD studies. This ranged from 5-7 hours per CHD model to print plus 1 hour for cleaning and dyeing (10); approximately 2 hours per TOF model segmentation and approximately 12 hours printing time (24). DeBoer et al claimed less than 12 hours for segmentation of an airway model (16). Gaisor reported that it took approximately 18 hours to print a 3D model demonstrating cloacal malformation (19).

Discussion

The 15 studies included in this review demonstrate objectively measured and user-reported benefit in using 3DP models in paediatric trainee and medical student training and educational settings in a

Confidential: For Review Only

range of subspecialties with a variety of outcomes. The quality of evidence, however, is diminished by a number of factors.

There are limitations to our review and sources of bias within the evidence base. Many studies were single centred, cross-sectional studies with a small number of study participants and no long-term follow-up which produce lower quality results according to validated tools of assessing medical educational studies (27). The exception was one study which recruited participants from the USA, South Korea, and Canada (10). One study from our search was in Chinese and an English translation of the full paper was not available, and was therefore excluded (28). Due to the heterogeneity in outcome measures we were not able to pool the results and directly compare the included studies as a meta-analysis, for instance.

A number of the studies did not take into account potential confounding factors. For instance, Smerling et al (15) measured the participants' self-reported understanding of CHDs immediately before and after exposure to 3DP CHD models. Each group entering the 3DP model station, however, had differing exposures depending on which group they were randomised to. Those who would have ended the workshop with the 3DP station would have watched an embryology video, attended a lecture on CHDs and examined pathology stations and may have reported greater confidence in understanding CHDs compared to those who started the workshop at the 3DP station.

The lack of randomisation, coupled with these studies not reporting how participants were recruited or randomised, is a source of selection bias. Some studies had failed to report baseline characteristics of participants (13,22) which means that the impact of using 3DP models may not be accurately reflected in outcome measurements. Cheng et al (13) had only analysed subjective

Confidential: For Review Only

outcome measures such as confidence in the pathology in focus, however objective measures were not scrutinised. Another source of bias in this study arises from the questions posed towards the control and intervention groups. Questions posed to the control group included "I understand the anatomy…" and "my training has fully prepared me for performing…", whilst those in the intervention group were asked whether "…the model improved my understanding …" and "the model was a valuable training tool…". Wording inconsistencies may cause the questions to become leading.

In an educational setting, the reporting of knowledge retention and translatability of knowledge and skill retention is key. Despite this, only one study had reported long-term outcomes, which had shown that there was no significant difference after at least 2-months in terms of flexible bronchoscopy performance on a testing apparatus (16). No study had assessed if training procedures with 3DP had led to improved clinical outcomes. Outcome measures such as time taken to complete procedures in a clinical setting or procedural success/complication rates have been considered in studies focussing on simulation in medical education (29,30). It has also been demonstrated that using 3DP models for pre-operative planning have reduced operating theatre times by up to an hour (31). There is potential for future paediatric studies to evaluate the clinical translation of 3DP model utility in training.

Ten studies were based upon CHD, possibly due to CHDs being the most common birth defect (32). Traditional teaching of CHD has been delivered using 2D diagrams (for example, in textbooks), cross-sectional imaging modalities (MRI, CT, echocardiography), cadaveric specimens and off-the-shelf models (33). However, these tools pose a variety of problems, including difficulty in translating planar images into 3D reconstructions in the learner's mind and lack of tactile feedback in the case of imaging, and lack of availability of specific pathologies in the case of cadaveric specimens and

Confidential: For Review Only

plastic models. The subgroup of studies focussing on CHD have shown to overcome these issues, and have demonstrated increased understanding with more complex models (15). 3DP models have shown their strengths in simplifying complex concepts which has typically proven to be challenging with congenital defects (34) and therefore can be a great educational tool to implement into routine practice. This is especially useful for adult clinicians who will be involved in transition-to-adult-services to provide care for the growing population of adults with congenital diseases (35,36). As CHD proves to be difficult to those with minimal training (34) it would be beneficial for future studies assessing the use of 3DP models in non-paediatric clinicians.

When considering the accuracy of models for procedure simulation or teaching, the model's size/shape and the suitability of the material used should be considered. The accuracy of model reconstruction has been demonstrated previously (37). In terms of material, acrylonitrile butadiene styrene was shown to mimic bone the most when used with a drill and craniotomy (13). However, replicating the consistency of soft tissue such as myocardium has been shown to be challenging (10). Whilst the motions of performing surgical techniques on a 1:1 scale model would be technically useful, the difference in material compliance would detract from the overall surgical experience. Another challenge would be the lack of surrounding structures and the inability to replicate bleeds or leaks, such as the of dural structures and cerebrospinal fluid (13). However, it has been demonstrated that soft tissues and artificial blood can be introduced to 3DP simulation models to replicate real patient anatomy and physiology (38). Whilst increasing the complexity of the models, improving accessibility and user-interface with 3D segmentation could ease their implementation in future paediatric simulation sessions.

Several reviews have explored the use of 3DP models within several other disciplines of medicine, namely undergraduate anatomy education and surgical training. In studies using 3DP models

Confidential: For Review Only

compared to lectures, textbooks and 2D radiological imaging there were statistically significant improvement in medical student performance on anatomy tests. Users of 3DP models reported great utility as an educational tool for teaching anatomy (39–41). As a tool for surgical trainees, participants using 3DP models reported greater confidence in understanding surgical pathologies and intra-operative confidence in performing procedures (42,43).These systematic reviews had also identified that many of these studies had non-randomised cohorts, small sample sizes, with inconsistencies in measuring or reporting outcomes (such as differing pre- and post-test formatting or omission of certain measurements). The limitations from the studies reviewed in the aforementioned systematic reviews and from the studies in this current review highlights this common issue and should prompt future educational studies using 3DP to attempt to overcome these study design flaws.

Paediatric trainees in Low- and Middle-Income Countries (LMICs) face problems with lack of training opportunities due to lack of resources used traditionally to teach anatomy and pathophysiology (3,44). 3DP models may have a role in improving access to training resources in such countries. DeBoer and colleagues highlight their $2500 3DP training model costed 43-250% less than other commercially available trainers at time of publication, with simple maintenance requirements (16). 3DP models also confer the benefit of patient-specific pathologies that are seen in real-word clinical settings, as opposed to off-the-shelf models based upon generic anatomical structures which may not reflect real patient anatomy and pathological processes. Furthermore, the nature of the medium is highly conducive to collaboration. Using 3DP medical aids has shown benefit for patients in Sierra Leone (45), however there is a paucity of evidence in the use of the technology in paediatric teaching and training in LMICs, which would warrant further investigation into its cost-effectiveness.

Confidential: For Review Only

The current evidence base demonstrates a positive impact made by 3DP models on paediatric education and training objectively and subjectively for medical students and clinical trainees. The majority of studies failed to address sources of bias, assess clinically translatable outcomes, or evaluate the long-term benefits. Future studies should recruit larger cohorts, ideally at a multicentre level, randomise participants and adopt objective outcome measurements (e.g. anonymous anatomy examinations and monitoring of trainee clinical performance), including follow-up to assess long-term benefits. 3DP simulations also have the potential to be more sophisticated to imitate real scenarios with surrounding soft tissue structures and blood.

References

1. Royal College of Paediatrics and Child Health. RCPCH position paper in response to the Shape of Training Review. 2017 Oct.
2. Bindal T, Wall D, Goodyear HM. Medical students' views on selecting paediatrics as a career choice. Eur J Pediatr. 2011;170(9).
3. Sohail AH, Maan MHA, Sachal M, Soban M. Challenges of training and delivery of pediatric surgical services in developing economies: a perspective from Pakistan. BMC Pediatr. 2019 Dec 16;19(1).
4. Al-Yassin A, Long A, Sharma S, May J. How can general paediatric training be optimised in highly specialised tertiary settings? Twelve tips from an interview-based study of trainees. BMJ Paediatr Open. 2017 Sep 4;1(1).
5. Hilliard R, Bannister SL, Amin H, Baird B. Paediatric medical education: Challenges and new developments. Paediatr Child Health. 2009 May 1;14(5).
6. Regitz-Zagrosek V, Roos-Hesselink JW, Bauersachs J, Blomström-Lundqvist C, Cífková R, De Bonis M, et al. 2018 ESC Guidelines for the management of cardiovascular diseases during pregnancy. Eur Heart J. 2018 Sep 7;39(34).

Confidential: For Review Only

7. Lichtenberger JP, Tatum PS, Gada S, Wyn M, Ho VB, Liacouras P. Using 3D Printing (Additive Manufacturing) to Produce Low-Cost Simulation Models for Medical Training. Mil Med [Internet]. 2018 Mar 1;183(suppl_1):73–7. Available from: https://academic.oup.com/milmed/article/183/suppl_1/73/4959929

8. Ganguli A, Pagan-Diaz GJ, Grant L, Cvetkovic C, Bramlet M, Vozenilek J, et al. 3D printing for preoperative planning and surgical training: a review. Biomed Microdevices [Internet]. 2018 Sep 4;20(3):65. Available from: http://link.springer.com/10.1007/s10544-018-0301-9

9. Giannopoulos AA, Mitsouras D, Yoo S-J, Liu PP, Chatzizisis YS, Rybicki FJ. Applications of 3D printing in cardiovascular diseases. Nat Rev Cardiol [Internet]. 2016 Dec 27;13(12):701–18. Available from: http://www.nature.com/articles/nrcardio.2016.170

10. Yoo SJ, Spray T, Austin EH, Yun TJ, van Arsdell GS. Hands-on surgical training of congenital heart surgery using 3-dimensional print models. J Thorac Cardiovasc Surg [Internet]. 2017;153(6):1530–40. Available from: http://dx.doi.org/10.1016/j.jtcvs.2016.12.054

11. Moher D, Liberati A, Tetzlaff J, Altman DG. Preferred Reporting Items for Systematic Reviews and Meta-Analyses: The PRISMA Statement. PLoS Med [Internet]. 2009 Jul 21;6(7):e1000097. Available from: https://dx.plos.org/10.1371/journal.pmed.1000097

12. Beller EM, Glasziou PP, Altman DG, Hopewell S, Bastian H, Chalmers I, et al. PRISMA for Abstracts: Reporting Systematic Reviews in Journal and Conference Abstracts. PLoS Med [Internet]. 2013 Apr 9;10(4):e1001419. Available from: https://dx.plos.org/10.1371/journal.pmed.1001419

13. Cheng D, Yuan M, Perera I, O'Connor A, Evins AI, Imahiyerobo T, et al. Developing a 3D composite training model for cranial remodeling. J Neurosurg Pediatr. 2019;24(6):632–41.

14. Su W, Xiao Y, He S, Huang P, Deng X. Three-dimensional printing models in congenital heart disease education for medical students: A controlled comparative study. BMC Med Educ. 2018;18(1):1–6.

15. Smerling J, Marboe CC, Lefkowitch JH, Pavlicova M, Bacha E, Einstein AJ, et al. Utility of 3D

Confidential: For Review Only

Printed Cardiac Models for Medical Student Education in Congenital Heart Disease: Across a Spectrum of Disease Severity. Pediatr Cardiol [Internet]. 2019;40(6):1258–65. Available from: https://doi.org/10.1007/s00246-019-02146-8

16. Deboer EM, Wagner J, Kroehl ME, Albietz J, Shandas R, Deterding RR, et al. Three-Dimensional Printed Pediatric Airway Model Improves Novice Learners' Flexible Bronchoscopy Skills with Minimal Direct Teaching from Faculty. Simul Healthc. 2018;13(4):284–8.

17. Cromeens BP, Ray WC, Hoehne B, Abayneh F, Adler B, Besner GE. Facilitating surgeon understanding of complex anatomy using a three-dimensional printed model. J Surg Res [Internet]. 2017;216:18–25. Available from: http://dx.doi.org/10.1016/j.jss.2017.04.003

18. Costello JP, Olivieri LJ, Su L, Krieger A, Alfares F, Thabit O, et al. Incorporating three-dimensional printing into a simulation-based congenital heart disease and critical care training curriculum for resident physicians. Congenit Heart Dis. 2015;10(2):185–90.

19. Gasior AC, Reck C, Lane V, Wood RJ, Patterson J, Strouse R, et al. Transcending Dimensions: a Comparative Analysis of Cloaca Imaging in Advancing the Surgeon's Understanding of Complex Anatomy. J Digit Imaging. 2019;32(5):761–5.

20. Biglino G, Capelli C, Koniordou D, Robertshaw D, Leaver LK, Schievano S, et al. Use of 3D models of congenital heart disease as an education tool for cardiac nurses. Congenit Heart Dis. 2017;12(1):113–8.

21. D. P. Utility of 3 dimensional printing of hearts with complex congenital heart disease in the education of pediatric trainees and sonographers. FASEB J [Internet]. 2016;30(Meeting Abstracts):5–7. Available from: http://www.fasebj.org/content/30/1_Supplement/787.7.abstract?sid=a4993bad-734d-447f-a363-e524a7c6dee2%0Ahttp://ovidsp.ovid.com/ovidweb.cgi?T=JS&PAGE=reference&D=emed17&NEWS=N&AN=72317314

Confidential: For Review Only

22. Costello JP, Olivieri LJ, Krieger A, Thabit O, Marshall MB, Yoo SJ, et al. Utilizing Three-Dimensional Printing Technology to Assess the Feasibility of High-Fidelity Synthetic Ventricular Septal Defect Models for Simulation in Medical Education. World J Pediatr Congenit Hear Surg. 2014;5(3):421–6.

23. Hadden R, Grover S, Chuen J C-BJ. Assessing the Utility of 3D Printed Models of Mullerian Tract Anomalies for Clinical Education. Aust NEW Zeal J Obstet Gynaecol. 2018;58:55.

24. Loke YH, Harahsheh AS, Krieger A, Olivieri LJ. Usage of 3D models of tetralogy of Fallot for medical education: Impact on learning congenital heart disease. BMC Med Educ. 2017;17(1):1–8.

25. Jones TW, Seckeler MD. Use of 3D models of vascular rings and slings to improve resident education. Congenit Heart Dis. 2017;12(5):578–82.

26. White SC, Sedler J, Jones TW, Seckeler M. Utility of three-dimensional models in resident education on simple and complex intracardiac congenital heart defects. Congenit Heart Dis. 2018;13(6):1045–9.

27. Reed DA, Cook DA, Beckman TJ, Levine RB, Kern DE, Wright SM. Association Between Funding and Quality of Published Medical Education Research. JAMA [Internet]. 2007 Sep 5;298(9):1002. Available from: http://jama.jamanetwork.com/article.aspx?doi=10.1001/jama.298.9.1002

28. Zhang J, Zhang FZ, Hao JS, Chen M, Zheng J, Ni X. [The application of 3D simulationtemporal-bone-system for temporal bone anatomy training]. Lin Chung Er Bi Yan Hou Tou Jing Wai Ke Za Zhi [Internet]. 2016 Aug 20;30(16):1325–8. Available from: http://www.ncbi.nlm.nih.gov/pubmed/29797982

29. Zendejas B, Brydges R, Wang AT, Cook DA. Patient outcomes in simulation-based medical education: A systematic review. Journal of General Internal Medicine. 2013.

30. Griswold-Theodorson S, Ponnuru S, Dong C, Szyld D, Reed T, McGaghie WC. Beyond the Simulation Laboratory. Acad Med [Internet]. 2015 Nov;90(11):1553–60. Available from:

Confidential: For Review Only

http://journals.lww.com/00001888-201511000-00032

31. Tack P, Victor J, Gemmel P, Annemans L. 3D-printing techniques in a medical setting: a systematic literature review. Biomed Eng Online [Internet]. 2016 Dec 21;15(1):115. Available from: http://biomedical-engineering-online.biomedcentral.com/articles/10.1186/s12938-016-0236-4

32. van der Linde D, Konings EEM, Slager MA, Witsenburg M, Helbing WA, Takkenberg JJM, et al. Birth Prevalence of Congenital Heart Disease Worldwide. J Am Coll Cardiol [Internet]. 2011 Nov;58(21):2241–7. Available from: https://linkinghub.elsevier.com/retrieve/pii/S0735109711030798

33. Seslar SP, Shepard CW, Giroud JM, Aiello VD, Cook AC, Spicer DE, et al. Lost treasures: a plea for the systematic preservation of cadaveric heart specimens through three-dimensional digital imaging. Cardiol Young [Internet]. 2015 Dec 16;25(8):1457–9. Available from: https://www.cambridge.org/core/product/identifier/S1047951115002589/type/journal_article

34. Webb CL, Jenkins KJ, Karpawich PP, Bolger AF, Donner RM, Allen HD, et al. Collaborative Care for Adults With Congenital Heart Disease. Circulation [Internet]. 2002 May 14;105(19):2318–23. Available from: https://www.ahajournals.org/doi/10.1161/01.CIR.0000017557.24261.A7

35. Viner RM. Transition of care from paediatric to adult services: one part of improved health services for adolescents: Figure 1. Arch Dis Child [Internet]. 2008 Feb;93(2):160–3. Available from: https://adc.bmj.com/lookup/doi/10.1136/adc.2006.103721

36. Marelli AJ, Mackie AS, Ionescu-Ittu R, Rahme E, Pilote L. Congenital Heart Disease in the General Population. Circulation [Internet]. 2007 Jan 16;115(2):163–72. Available from: https://www.ahajournals.org/doi/10.1161/CIRCULATIONAHA.106.627224

37. Schievano S, Migliavacca F, Coats L, Khambadkone S, Carminati M, Wilson N, et al. Percutaneous Pulmonary Valve Implantation Based on Rapid Prototyping of Right Ventricular Outflow Tract and Pulmonary Trunk from MR Data. Radiology [Internet]. 2007

Confidential: For Review Only

Feb;242(2):490–7. Available from: http://pubs.rsna.org/doi/10.1148/radiol.2422051994

38. O’Reilly MK, Reese S, Herlihy T, Geoghegan T, Cantwell CP, Feeney RNM, et al. Fabrication and assessment of 3D printed anatomical models of the lower limb for anatomical teaching and femoral vessel access training in medicine. Anat Sci Educ [Internet]. 2016 Jan;9(1):71–9. Available from: http://doi.wiley.com/10.1002/ase.1538

39. Yuen J. What Is the Role of 3D Printing in Undergraduate Anatomy Education? A Scoping Review of Current Literature and Recommendations. Med Sci Educ [Internet]. 2020 Sep 3;30(3):1321–9. Available from: http://link.springer.com/10.1007/s40670-020-00990-5

40. Fleming C, Sadaghiani MS, Stellon MA, Javan R. Effectiveness of Three-Dimensionally Printed Models in Anatomy Education for Medical Students and Resident Physicians: Systematic Review and Meta-Analysis. J Am Coll Radiol [Internet]. 2020 Oct;17(10):1220–9. Available from: https://linkinghub.elsevier.com/retrieve/pii/S1546144020305561

41. Ye Z, Dun A, Jiang H, Nie C, Zhao S, Wang T, et al. The role of 3D printed models in the teaching of human anatomy: a systematic review and meta-analysis. BMC Med Educ [Internet]. 2020 Dec 29;20(1):335. Available from: https://bmcmededuc.biomedcentral.com/articles/10.1186/s12909-020-02242-x

42. Langridge B, Momin S, Coumbe B, Woin E, Griffin M, Butler P. Systematic Review of the Use of 3-Dimensional Printing in Surgical Teaching and Assessment. J Surg Educ [Internet]. 2018 Jan;75(1):209–21. Available from: https://linkinghub.elsevier.com/retrieve/pii/S1931720417301836

43. Vakharia VN, Vakharia NN, Hill CS. Review of 3-Dimensional Printing on Cranial Neurosurgery Simulation Training. World Neurosurg [Internet]. 2016 Apr;88:188–98. Available from: https://linkinghub.elsevier.com/retrieve/pii/S1878875015017350

44. Cass H, Barclay S, Gerada C, Lumsden DE, Sritharan K. Complexity and challenge in paediatrics: a roadmap for supporting clinical staff and families. Arch Dis Child [Internet]. 2019 Jun 11;archdischild-2018-315818. Available from:

Confidential: For Review Only

https://adc.bmj.com/lookup/doi/10.1136/archdischild-2018-315818

45. van der Stelt M, Verhulst AC, Vas Nunes JH, Koroma TAR, Nolet WWE, Slump CH, et al. Improving Lives in Three Dimensions: The Feasibility of 3D Printing for Creating Personalized Medical Aids in a Rural Area of Sierra Leone. Am J Trop Med Hyg [Internet]. 2020 Apr 1;102(4):905–9. Available from: http://www.ajtmh.org/content/journals/10.4269/ajtmh.19-0359

Funding statement

The authors received no specific grant from any funding agency in the public, commercial or not-for-profit sectors for conducting this review. The authors acknowledge the generous support of the British Heart Foundation (CH/17/1/32804) and The Grand Appeal (Bristol Children's Hospital Charity).

Competing interests statement

The authors declare that the research was conducted in the absence of any commercial or financial relationships that could be construed as a potential conflict of interest.

Patient consent for publication

Not required.

Ethics approval

Ethical approval was not required as no primary patient data was used in this review.

Confidential: For Review Only

Data availability statement

All data relevant to the study are included in the article or uploaded as supplemental information.

No additional information.

Author contributions

Ashar Asif conceived the study and drafted the study protocol, registered the review to Prospero, conducted the review searches, was first reviewer of the eligible studies and lead the writing of the manuscript.

Elgin Meng Lee reviewed eligible papers and contributed to the writing of the manuscript.

Massimo Caputo assisted in editing the final manuscript.

Giovanni Biglino assisted in editing the final manuscript.

Andrew Shearn conceived and supervised the study, was adjudicator for any conflicts in study selection for the review, and assisted in editing the final manuscript.

All authors have read and approvedf the final manuscript.

Confidential: For Review Only

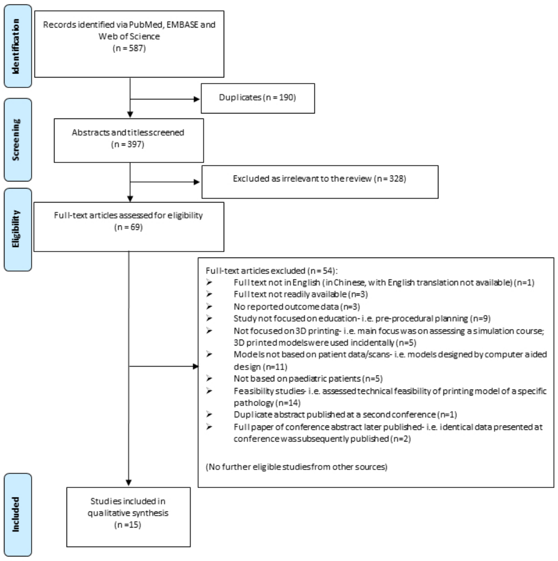
Identification
Records identified via PubMed, EMBASE and Web of Science (n = 587)
Duplicates (n = 190)
Screening
Abstracts and titles screened (n = 397)
Excluded as irrelevant to the review (n = 328)
Eligibility
Full-text articles assessed for eligibility (n = 69)
Full-text articles excluded (n = 54):
Full text not in English (in Chinese, with English translation not available) (n=1)
Full text not readily available (n=3)
No reported outcome data (n=3)
Study not focused on education- i.e. pre-procedural planning (n=9)
Not focused on 3D printing- i.e. main focus was on assessing a simulation course; 3D printed models were used incidentally (n=5)
Models not based on patient data/scans- i.e. models designed by computer aided design (n=11)
Not based on paediatric patients (n=5)
Feasibility studies- i.e. assessed technical feasibility of printing model of a specific pathology (n=14)
Duplicate abstract published at a second conference (n=1)
Full paper of conference abstract later published- i.e. identical data presented at conference was subsequently published (n=2)
(No further eligible studies from other sources)
Included
Studies included in qualitative synthesis (n =15)

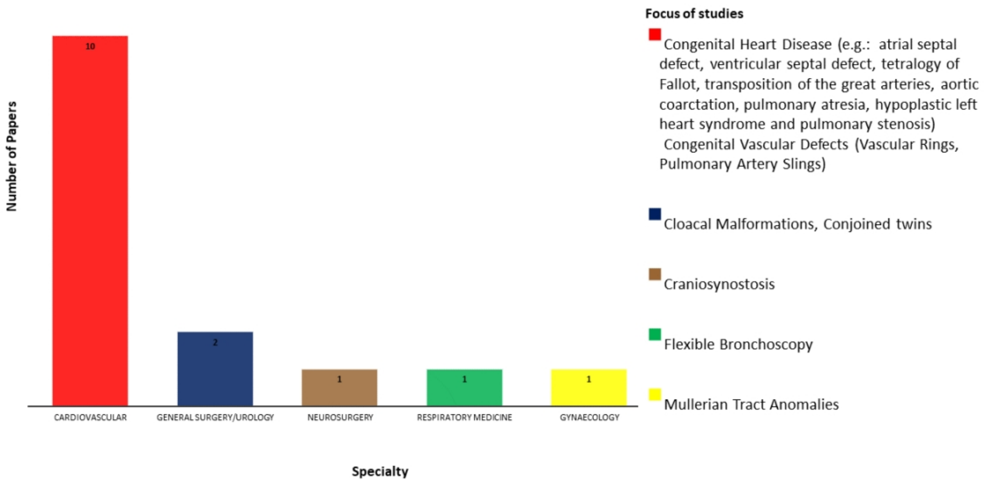
Number of Papers
10
2
1
1
1
CARDIOVASCULAR
GENERAL SURGERY/UROLOGY
NEUROSURGERY
RESPIRATORY MEDICINE
GYNAECOLOGY
Specialty
Focus of studies
Congenital Heart Disease (e.g.: atrial septal defect, ventricular septal defect, tetralogy of Fallot, transposition of the great arteries, aortic coarctation, pulmonary atresia, hypoplastic left heart syndrome and pulmonary stenosis)
Congenital Vascular Defects (Vascular Rings, Pulmonary Artery Slings)
Cloacal Malformations, Conjoined twins
Craniosynostosis
Flexible Bronchoscopy
Mullerian Tract Anomalies

BMJ Paediatrics Open

The Role of 3D Printing Technology in Paediatric Teaching and Training: a Systematic Review

Journal:	*BMJ Paediatrics Open*
Manuscript ID	bmjpo-2021-001050.R2
Article Type:	Original research
Date Submitted by the Author:	14-May-2021
Complete List of Authors:	Asif, Ashar; University of Bristol Faculty of Medicine and Dentistry,<br>Lee, Elgin; Newcastle Upon Tyne Hospitals NHS Foundation Trust,<br>Caputo, Massimo; University of Bristol Faculty of Medicine and Dentistry; University of Bristol School of Clinical Science, Bristol Heart Institute<br>Biglino, Giovanni; University of Bristol School of Clinical Science, Bristol Heart Institute; Imperial College London National Heart and Lung Institute<br>Shearn, Andrew; University of Bristol Faculty of Medicine and Dentistry; University of Bristol School of Clinical Science, Bristol Heart Institute
Keywords:	Data Collection, Information Technology, Technology, Cardiology, Neurosurgery

SCHOLARONE™
Manuscripts

I, the Submitting Author has the right to grant and does grant on behalf of all authors of the Work (as defined in the below author licence), an exclusive licence and/or a non-exclusive licence for contributions from authors who are: i) UK Crown employees; ii) where BMJ has agreed a CC-BY licence shall apply, and/or iii) in accordance with the terms applicable for US Federal Government officers or employees acting as part of their official duties; on a worldwide, perpetual, irrevocable, royalty-free basis to BMJ Publishing Group Ltd ("BMJ") its licensees and where the relevant Journal is co-owned by BMJ to the co-owners of the Journal, to publish the Work in this journal and any other BMJ products and to exploit all rights, as set out in our licence.

The Submitting Author accepts and understands that any supply made under these terms is made by BMJ to the Submitting Author unless you are acting as an employee on behalf of your employer or a postgraduate student of an affiliated institution which is paying any applicable article publishing charge ("APC") for Open Access articles. Where the Submitting Author wishes to make the Work available on an Open Access basis (and intends to pay the relevant APC), the terms of reuse of such Open Access shall be governed by a Creative Commons licence – details of these licences and which Creative Commons licence will apply to this Work are set out in our licence referred to above.

Other than as permitted in any relevant BMJ Author's Self Archiving Policies, I confirm this Work has not been accepted for publication elsewhere, is not being considered for publication elsewhere and does not duplicate material already published. I confirm all authors consent to publication of this Work and authorise the granting of this licence.

Confidential: For Review Only

The Role of 3D Printing Technology in Paediatric Teaching and Training: a Systematic Review

Ashar Asif[1], ORCiD 0000-0002-9361-1083

Elgin Meng Lee[2] ORCiD 0000-0002-0054-1574

Massimo Caputo[1, 3] ORCiD 0000-0001-7508-0891

Giovanni Biglino[3, 4] ORCiD 0000-0003-0413-149X

Andrew I. U. Shearn[1, 3] ORCiD 0000-0002-3741-9118

1. Bristol Medical School, University of Bristol, Bristol Royal Infirmary, Bristol, UK, BS2 8HW,

2. Newcastle upon Tyne Hospitals NHS Foundation Trust, Freeman Road, Newcastle upon Tyne, UK, NE7 7DN

3. Bristol Heart Institute, Bristol Royal Infirmary, Bristol, UK, BS2 8HW

4. National Heart and Lung Institute, Guy Scadding Building, Imperial College London, London, UK, SW3 6LY

Corresponding author

Dr Andrew Shearn

University of Bristol

Research Floor Level 7

Bristol Royal Infirmary

Upper Maudlin Street

Bristol BS2 8HW

Confidential: For Review Only

Tel: (44) 0117 3423142

Email: andrew.shearn@bristol.ac.uk

Word count: 2597

Abstract

Background

In the United Kingdom, undergraduate paediatric training is brief resulting in trainees with a lower paediatric knowledge base compared to other aspects of medicine. With congenital conditions being successfully treated at childhood, adult clinicians encounter and will need to understand these complex pathologies. Patient-specific 3D printed (3DP) models have been used in clinical training, especially for rarer, complex conditions. We perform a systematic review to evaluate the evidence base in using 3DP models to train paediatricians, surgeons, medical students, and nurses.

Methods

Online databases PubMed, Web of Science and EMBASE and were searched between January 2010 and April 2020 using search terms relevant to “paediatrics”, “education”, “training” and “3D printing”. Participants were medical students, postgraduate trainees or clinical staff. Comparative studies (patient-specific 3DP models vs. traditional teaching methods) and non-comparative studies were included. Outcomes gauged objective and subjective measures: test scores, time taken to complete tasks, self-reported confidence and personal preferences on 3DP models. If reported, the cost of, and time taken, to produce the models were noted.

Confidential: For Review Only

Results

From 587 results, 15 studies fit the criteria of the review protocol, with 5/15 being randomised-control studies and 10/15 focussing on cardiovascular conditions. Participants using 3DP models demonstrated improved test scores and, faster times to complete procedures and identify anatomical landmarks compared to traditional teaching methods (2D diagrams, lectures, videos, supervised clinical events). User feedback was positive, reporting greater user self-confidence in understanding concepts with users wishing for integrated use of 3DP in regular teaching. Four studies reported the costs and times of production, which varied depending on model complexity and printer. 3DP models were cheaper than "off-the-shelf" models available on the market and had the benefit of utilising real-world pathologies. These, mostly non-randomised and single-centred, studies did not address bias or report long-term or clinically translatable outcomes.

Conclusions

3DP models were associated with greater user satisfaction and good short-term educational outcomes, with low quality evidence. Multi-centred, randomised studies with long-term follow-up and clinically assessed outcomes are needed to fully assess their benefits in this setting.

Systematic review registration

The review is registered on Prospero: CRD42020179656

https://www.crd.york.ac.uk/prospero/display_record.php?ID=CRD42020179656

Confidential: For Review Only

What is known about the subject

- Undergraduate paediatric education is known to be short and postgraduate paediatric training can be limited within certain regions.
- Patients with congenital diseases are surviving to adulthood, meaning adult clinicians are seeing an increase of cases which were traditionally considered as ‘paediatric conditions’ .
- Patient specific 3D printed models have been used in other medical and surgical specialties for hands-on education and training and can compensate for these pitfalls.

What this review adds

- 3D printed models in paediatric education and training sessions have been shown to improve immediate educational and procedural performance, with high user satisfaction.
- The quality of evidence is poor due to unaddressed confounding factors, small study cohorts and poor study design.
- In the few studies that reported costs, 3D printed models had the benefit of producing models at relatively lower costs compared to alternative resources.

Introduction

In 2017, the Royal College of Paediatrics and Child Health published a response to the “Shape of Training Review” of postgraduate training within the United Kingdom. They observed that “doctors coming into paediatric specialty training do so from a lower knowledge base than adult medicine” (1). This could partly be explained by the brevity of undergraduate exposure to paediatrics, typically 4-8 weeks during the entirety of medical school (2). Furthermore some paediatric trainees report a lack of educational opportunities within their postgraduate training programme (3), potentially driven by the varied facilities offered by different regions and centres (4,5).

Confidential: For Review Only

It is increasingly important for non-paediatric clinicians to fully appreciate paediatric pathologies. Novel therapies have allowed many children, who would have had poor outcomes from previously life-limiting diseases, to now live with such pathologies resulting in complex patients with chronic conditions (5). Clinical care and observation over the adult lifetime of these patients is often overseen by a myriad of clinicians, frequently requiring specialised oversight such as in the case of pregnant women with congenital heart disease (CHD) (6).

In recent years, 3D printing (3DP) has played a role in a variety of medical and surgical training and educational settings (7–9). 3DP models can replicate in-vivo clinical pathologies based on radiological images, allowing trainees to be exposed to pathologies and potential procedures they would not ordinarily experience through their routine clinical exposure (10). With this review we aim to evaluate the current evidence base regarding the utility of 3DP models compared to traditional teaching methods (i.e. 2D images/diagrams, lectures, videos, ward-based teaching) in educating and training of medical students and clinical trainees in paediatrics.

Methods

Study Protocol

The study protocol is registered on PROSPERO and can be found with the following ID: CRD42020179656. Throughout the review, the authors referred and adhered to the Preferred Reporting Items for Systematic reviews and Meta-Analyses (PRISMA) guidelines (11).

Literature search strategy

Confidential: For Review Only

The databases PubMed, Web of Science and EMBASE were searched. The search included all published material since January 2010 (last accessed in April 2020) in the English language. This timeframe was selected to ensure the studies reviewed used the most recent available technology. The following terms were used across all databases: ((3d-prin*) OR (3d prin*) OR (three-dimensional print*) OR (three dimensional print*) OR (3-d print*)) AND ((paediat*) OR (pediat*)) AND ((education) OR (teaching) OR (training) OR (session) OR (simulation)). Synonyms and spelling variations were also searched to allow for international spellings and alternative terms (e.g. paediatrics vs. pediatrics). Searches on PubMed included MeSH headings.

Selection criteria

The inclusion criteria for final review were studies using 3DP models derived from paediatric patient imaging data (e.g. computed tomography (CT), magnetic resonance imaging (MRI), ultrasound (US) or echocardiography) and that occurred in an educational or training setting. This was defined as a pre-planned session or programme as part of an educational syllabus, training programme or professional course separate from a clinical setting and not aimed to directly contribute towards patient outcomes. This excluded studies which used 3DP models to facilitate communication with patients, carers or clinical staff. Studies that produced a 3DP model for pre-operative planning for a specific case were also excluded. Included studies evaluated the use of patient-specific models with physicians, surgeons, nurses and allied health professionals undergoing teaching, training or simulation. Comparative studies would compare the use of 3DP models against traditional educational methods such as lectures, tutorials, practical sessions, or textbooks. Case reports and case series were also included if they used the models in an educational setting and reported educational outcome measures. Published conference abstracts were included as per PRISMA guidance to minimise publication bias (12). Primary outcome measures were either objective measures such as pre-/post-intervention testing scores or procedure performance, or subjective

Confidential: For Review Only

measures such as participant-reported opinions from questionnaires. Additional outcome measures that were recorded included the time taken to produce models and the cost of model production.

Data extraction and appraisal of evidence

The titles and abstracts of all papers obtained during the searches were screened for relevance by two authors independently (AA, EL). Relevant papers were then independently assessed for eligibility in full against the study protocol (AA, EL). Any disagreements were resolved by consensus, with the senior author (AS) acting as the final adjudicator for any unresolved discrepancies. The final, included papers were then reviewed and appraised by AA and EL. Data was extracted manually onto a Microsoft Excel spreadsheet.

Patient and Public Involvement

No patients were involved, nor was any patient data used, in the development or analysis of this review.

Results

Literature Search Outcomes

Our search resulted in a total of 587 articles. The screening for eligibility is illustrated in Figure 1. A final total of 15 articles were included, with no additional articles identified from cross-referencing.

Figure 1. PRISMA flowchart of identifying eligible studies.

Confidential: For Review Only

Characteristics of Selected Studies

The studies included in this review focused on five main specialties relating to paediatric or congenital disorders: Cardiovascular surgery, General Surgery, (1 study shared with Urology), Neurosurgery, Respiratory Medicine and Gynaecology. Figure 2 breaks down the included studies by subspecialty.

The majority of eligible studies (n=10) assessed the use of 3DP models in CHD. Out of the 15 included studies, 3 assessed the utility of 3DP models amongst undergraduates (13–15). One study was a multi-centre study (10) and only 2 studies followed up participants after a period of time (13,16). The majority of studies were non-randomised, single-centred cross-sectional studies with no sample size calculations to determine the optimal number of study participants. Tables 1, 2 and 3 summarise the study design, participants and findings of the included studies.

Figure 2. Breakdown of specialties for included studies

Confidential: For Review Only

Study	*Design and Objective*	*Study Cohort*	*Primary outcomes*	*Secondary outcomes*
Facilitating surgeon understanding of complex anatomy using a three-dimensional printed model (17).	Use of 3DP to improve understanding of complex anatomy (conjoined twins) vs CT scan and Digital Reconstruction.	21 (12 Paediatric Surgery Attendings, 9 Paediatric Surgery and General Surgery Residents).	3DP models improved scale and shape orientation, identification of anatomy, but not linear/point-to-point distances.<br>Not all outcome data were reported.<br>Using 3DP models was associated improved test times compared to CT alone and CT with digital reconstructions (6.6 min vs 18.9 min vs 14.9 min; p <0.05).	None reported.
Hands-on surgical training of congenital heart surgery using 3-dimensional print models (10).	Use of 3DP as part of surgical training for CHD. Participants were given a questionnaire following the surgical simulation session.	81 Cardiovascular surgery trainees and Attendings. Conducted in the USA, Canada and South Korea.	3DP models demonstrated necessary pathological findings, and were acceptable for surgical training. Materials used differed from real human myocardium.	Printing took 5-7 hours for each model at an estimated total cost of $150 to $210. The average cost for print materials per model was $60.
Incorporating Three-dimensional Printing into a Simulation-based Congenital Heart Disease and Critical Care Training Curriculum for Resident Physicians (18).	Use of 3DP models in a 60-minute simulation teaching VSD anatomy, echocardiography, repair, and postoperative critical care management. Participants were given a pre- and post-session test scored out of 10.	23 Paediatric Resident Physicians.	3DP models improved their knowledge acquisition 4.83 vs 7.33 p=0.0082), knowledge reporting (4.25 vs 6.86 p=0.01) and structural conceptualisation (4.17 vs 7.22 p<0.0001).	None reported
Transcending Dimensions: a Comparative Analysis of Cloaca Imaging in Advancing the Surgeon's Understanding of Complex Anatomy (19).	To compare the effectiveness of 4 different modalities in teaching cloacal malformations in the context of operative planning (2D contrast study cloacagram vs 3D rotatable CT scan reconstruction vs software-enhanced 3D video animation vs 3DP cloaca model).	59 paediatric surgeons (29 trainees and 30 Attendings).	Participants using 3DP models scored significantly better (p<0.001) compared to those using other modalities. 2D cloacogram vs 3D CT vs enhanced 3D CT vs 3DP; Trainees- 10.5% vs 46.7% vs 67.1% vs 73.8%; Attendings- 22.2%, 54.8%, 66.2%, 74.0%.	18 hours to print. Cost of models not reported.
Use of 3D models of congenital heart disease as an education tool for cardiac nurses (20).	Use of 3DP models to improve knowledge in cardiac nurses of various CHD after treatment: tetralogy of Fallot, transposition of the great arteries, aortic coarctation, pulmonary atresia, hypoplastic left heart syndrome. Participants were given a 5-question survey with Likert questions.	100 cardiac nurses (65 paediatric, 35 adult).	Percentage of participants that agree/strongly agree that 3DP models:<br>• improved their learning experience: 60%,<br>• improved understanding of anatomy: 86%<br>• provided spatial orientation: 70%<br>• displayed anatomical complexity after treatment: 66%,	None reported.

Confidential: For Review Only

			• provided more information than diagrams: 74%.<br>Mean score for utility: 5.1 out of 7.<br>Participants reported the value of being able to see inside the model heart explicitly and potential of using 3DP models in education/training.	
Utility of 3D Printed Cardiac Models for Medical Student Education in Congenital Heart Disease: Across a Spectrum of Disease Severity (15).	The impact of 3D printed models for PS, ASD, aortic coarctation, TOF, TGA, HLHS. A 4-station workshop used 2D images, embryology videos, spoken explanation, pathology specimens and 3DP models. Students rotated between all stations. They were given a pre-station and post-station questionnaire at the 3D printed station assessing self-reported confidence and ranked the different teaching modalities.	45 first year medical students.	Using 3DP models improved self-reported confidence scores (PS (0.3, p< 0.001), ASD (0.6, p< 0.001), TOF (0.8, p< 0.001), d-TGA (0.9, p< 0.001), Coarct (0.8, p <0.001), HLHS (1.1, p< 0.001)).<br>Strong correlation between the complexity of pathology and perceived knowledge increase (R^2 = 0.73, p=0.03).<br>Participants agreed that they would use 3DP models in future teaching sessions (mean score: 4.40 out of 5).<br>74.2% of participants scored 3DP models at least 3 out of 5 for utility as an educational tool.	None reported.
Utility of 3-Dimensional Printing of Hearts with Complex Congenital Heart Disease in the Education of Pediatric Trainees and Sonographers† (21).	Use of 3DP models vs CT/MRI imaging to improve general understanding of CHD.	10 (5 Paediatric Residents, 2 Sonographers and 2 Cardiology Fellows).	80% of residents and 100% of sonographers and fellows felt that their understanding of CHD improved with 3DP models and felt that these should be utilised in the education of paediatric trainees.	None reported.
Utilizing Three-Dimensional Printing Technology to Assess the Feasibility of High-Fidelity Synthetic Ventricular Septal Defect Models for Simulation in Medical Education (22).	Teaching and simulation using 3DP CHD models, including instruction on surgical incisions and suturing skills. Score out of 10.	29 (16 Medical and 13 Pre-medical students).	Significant (p<0.0001) pre- and post-seminar score improvement in knowledge acquisition (3.22 vs 7.02), knowledge reporting (2.16 vs 6.60), and structural conceptualization (2.17 vs 6.31) of VSD.	None reported.

Table 1. Study characteristics of cross-sectional studies. †Conference abstract. VSD: ventricular septal defect, TOF: Tetralogy of Fallot, ASD: atrial septal defect, HLHS: hypoplastic left heart syndrome, TGA; transposition of the great arteries, PS: pulmonic stenosis, CHD: congenital heart disease.

Confidential: For Review Only

Study	Design and Objective	Study Cohort	Primary outcomes	Secondary outcomes
Assessing the utility of 3D Printed models of Mullerian tract anomalies for clinical education† (23).	Impact of 3DP models on trainees' understanding of Mullerian tract anomalies and assessment of the feasibility of producing such models	Gynaecologists and general surgery trainees (exact number of participants not given).	3DP models were found to increase gynaecologists' understanding of Mullerian tract anomalies and their confidence in surgery to correct them.	None reported.
Developing a 3D composite training model for cranial remodelling (13).	Use of 3DP models at 2 annual practical courses to teach neurosurgical techniques (FOA and ES) for correction of craniosynostosis. Surveys were given to attendees and non-attendees a year after the course.	33 Students, Resident, Fellows and Attending surgeons over 2 years.	Models were a valuable training tool for surgical techniques and improved understanding and preparedness to perform the procedures.	None reported.
Three-Dimensional Printed Pediatric Airway Model Improves Novice Learners' Flexible Bronchoscopy Skills with Minimal Direct Teaching From Faculty (16).	The impact of 3DP airway models in training/teaching Flexible Bronchoscopy to paediatric residents. Assessed on identifying 6 structures on bronchoscopy and the time taken to complete the task. Control and intervention groups were assessed pre-study, post-study and a minimum of 2 months following the session.	27 Paediatric Residents (PGY2) at the beginning of their Paediatric Respiratory rotation (18 in intervention group, 9 in control group).	Intervention vs control groups: median difference between pre- and post-study scores 4 vs 0 ($p<0.001$), median difference in times -432 seconds vs 0 seconds ($p<0.001$).<br><br>Minimum of 2 months post-study, intervention vs control: median pre-study and delayed post-study score difference (2.5 vs 1 ($p=0.123$)), median difference in pre- and delayed post-study times - 180 seconds vs 0 seconds ($p=0.141$).	Estimated total cost for materials and labour to trainer- $2,500. Cost of fused deposition modelling-type 3D printer- $32,000.<br><br>12 hours to produce the computer model ready to print.
Three-dimensional printing models in congenital heart	To compare knowledge acquisition and structural conceptualisation of 3 subtypes of	63 Medical students (32 in intervention group,	3DP models significantly improved subjective understanding (mean score for intervention and control groups, out of 100:	None reported.

Table 2. Study characteristics of cohort studies. †Conference abstract. FOA: Fronto-orbital Advancement, ES: Endoscopic Suturectomy.

Confidential: For Review Only

disease education for medical students: a controlled comparative study (14).	VSD for medical students with 3DP models vs without 3DP models. Participants were given a post-session test assessing knowledge acquisition and structural conceptualisation, and a subjective questionnaire.	31 in control).	72.19 vs 56.12 (p <0.0001)) and objective structural conceptualization (mean score for intervention and control groups, out of 30: 18.44 vs 14.52 (p=0.03)) but not in knowledge acquisition of VSD (mean score for intervention and control groups, out of 70: 44.06 vs 36.77 (p=0.06)).	
Usage of 3D models of tetralogy of Fallot for medical education: impact on learning congenital heart disease (24).	To compare conventional 2D drawings vs 3DP models in knowledge acquisition of TOF. Pre- and post-session knowledge tests (out of 9) were performed, and self-reported questionnaires (out of 25) were completed.	35 Paediatric Residents (17 in 2D image group, 18 in 3DP model group).	No observable significant difference between 3DP models and 2D images in terms of knowledge acquisition (mean post-test scores: 6.0 vs 6.3) or self-reported confidence in ToF (21 vs 20 (p=0.39)), but 3DP models provided significantly greater learner satisfaction (24 vs 21 (p=0.03)).	Cost to produce models ranged from $15 to $300 depending on the printer. Time taken to print each model was approximately 12 hours.
Use of 3D models of vascular rings and slings to improve resident education (25).	Block randomisation was employed to recruit participants.<br><br>The intervention group used of 3DP models in didactic teaching sessions to improve understanding of vascular rings and pulmonary artery slings. The control group were shown virtual models. Pre-session and post-session subjective questionnaires and knowledge testing were performed by participants.	36 Paediatric and Emergency Medicine Residents.	Both groups self-reported improved confidence in identifying, diagnosing and treating vascular rings and slings. Intervention groups scored significantly higher than the control groups (62.2% vs 45.1%; p=0.001), however the score improvement from pre-test to post-test scores was insignificant between intervention and control groups (2.6 vs 1.8; p=0.084).	None reported.
Utility of three-dimensional models in resident education on simple and complex intracardiac congenital heart defects (26).	Block randomisation was employed to recruit participants.<br><br>Both groups attended a lecture with 2D images/virtual models. The intervention group were given 3D printed models during the lecture as an adjunct to the lecture content. Pre-session and post-session subjective questionnaires and knowledge testing were performed by participants.	60 Paediatric and Emergency Medicine Residents (26 in the VSD portion of the study, and 34 in the TOF portion of the study).	Subjective reporting of confidence in understanding the pathology significantly increased in both control and intervention groups in the VSD and TOF portions of the study. In the VSD study, the control group had a significantly greater improvement in post-test scores compared to those in the intervention arm (3.16 vs 1.93; p=0.004). In the TOF study, despite the intervention arm having a greater post-session test score than the control group (6.06 vs 5.29; p=0.037), there was no significant difference in pre-session and post-session score changes between control and intervention groups (2.23 vs 2.65; p=0.406).	None reported.

Table 3. Study characteristics of randomised control trials. VSD: ventricular septal defect, TOF: Tetralogy of Fallot.

Confidential: For Review Only

Primary outcomes

Studies reported various objective and subjective (self-reported) outcome measures across a range of parameters, including knowledge acquisition, knowledge reporting, structural conceptualisation and identification, confidence in management and in explaining anatomy to others, learner satisfaction and increased subject interest. The key findings of each study are summarised in Tables 1-3.

Objective assessment of 3DP models in the setting of CHD generally reported observable improvement in knowledge acquisition, structural conceptualisation and greater improvement from baseline assessment scores compared to control groups (18,22,26). However, observable differences were not always statistically significant (14,24,25). Those who used 3DP models reported improved subject understanding, increased confidence in reporting the pathology and in identifying the pathology compared to controls (14,24–26). Participants felt that 3DP models were more useful than diagrams and videos and would like for these models to be implemented in their usual training (15,20,21). However, from a surgical standpoint, whilst useful in training and demonstrating procedures, the textural properties of the material used did not match that of myocardial tissue (10).

The studies in paediatric gynaecology and general surgery revealed better self-reported and objective measured outcomes when using 3DP models (17,19,23). Identification of abdominal organs and cloacal malformations were more accurate and performed faster in those in the intervention arm of the studies compared to the control arm (17,19). 3DP models left participants with greater self-reported confidence in understanding Mullerian tract anomalies (23).

Confidential: For Review Only

The two studies that followed-up participants assessed the impact of 3DP models on bronchoscopy and neurosurgical training (13,16). Those performing bronchoscopy on 3DP models compared to those who did not performed assessment tasks, such as marker identification and completion time, significantly better in immediate post-session testing. However these benefits were not noticeable on follow-up a minimum of 2 months after the initial session (16). Models produced for fronto-orbital advancement and endoscopic suturectomy simulation lead to improved understanding amongst attendees at the neurosurgical workshop, and at follow-up participants felt that they were more prepared compared to their colleagues who did not attend (13). This study, however, did not perform any quantitative analysis and did not objectively measure the impact 3DP models had at immediate or post-follow-up evaluation.

Cost and Duration of Model Generation

In four studies some information was given about the cost or the time taken to generate the 3DP models (10,16,19,24). Cost estimations varied widely, from $15 to $2500 depending on the type of printer, the material used, and whether other costs were included. Segmentation/model production time was also included for two of the CHD studies. In one study this was from 5-7 hours per CHD model to print, plus another 1 hour for cleaning and dyeing (10). In another study, it took approximately 2 hours per TOF model for segmentation and approximately 12 hours to print the model (24). DeBoer et al claimed less than 12 hours for segmentation of an airway model (16). Gaisor reported that it took approximately 18 hours to print a 3D model demonstrating cloacal malformation (19).

Discussion

Confidential: For Review Only

The 15 studies included in this review demonstrate objectively-measured and user-reported benefit in using 3DP models in paediatric trainee and medical student training and educational settings in a range of subspecialties, with a variety of outcomes. The quality of the evidence, however, is diminished by a number of factors.

There are limitations to our review and sources of bias within the evidence base. Many studies were single-centre, cross-sectional studies with a small number of study participants and no long-term follow-up, which produce lower quality results according to validated tools of assessing medical educational studies (27). The exception was one study which recruited participants from the USA, South Korea, and Canada (10). One study from our search was in Chinese and an English translation of the full paper was not available – it was therefore excluded (28). Due to the heterogeneity in outcome measures we were not able to pool the results and directly compare the included studies, for example as a meta-analysis.

A number of the studies did not take into account potential confounding factors. For instance, Smerling et al (15) measured the participants' self-reported understanding of CHDs immediately before and after exposure to 3DP CHD models. Each group entering the 3DP model station, however, had differing exposures depending on which group they were randomised to. Those who ended the workshop with the 3DP station would have previously watched an embryology video, attended a lecture on CHDs and examined pathology stations and hence may have reported greater confidence in understanding of CHDs compared to those who started the workshop at the 3DP station.

Confidential: For Review Only

The lack of randomisation, coupled with studies not reporting how participants were recruited or randomised, is a source of selection bias. Some studies do not report baseline characteristics of participants (13,22), which means that the impact of using 3DP models may not be accurately reflected in outcome measurements. Cheng et al (13) only analysed subjective outcome measures such as confidence in understanding the pathology in focus, however objective measures were not scrutinised. Another source of bias in this study arises from the questions posed to the control and intervention groups. Questions to the control group included, “I understand the anatomy…,” and, “My training has fully prepared me for performing…” However, those in the intervention group were asked whether, “…the model improved my understanding …,” and, “the model was a valuable training tool…” Wording inconsistencies may cause the questions to become leading.

In an educational setting, the reporting of knowledge retention and translatability of knowledge and skill retention is key. Despite this, only one study reported long-term outcomes, which showed that there was no significant difference in the performance of participants on a testing apparatus for flexible bronchoscopy when followed-up a minimum of 2 months after the original training session (16). No study assessed if training procedures with 3DP led to improved clinical outcomes. Outcome measures such as time taken to complete procedures in a clinical setting or procedural success/complication rates have been considered in studies focussing on simulation in medical education (29,30). It has also been demonstrated that using 3DP models for pre-operative planning reduced operating theatre times by up to an hour (31). There is potential for future paediatric studies to evaluate the clinical translation of 3DP model use in training.

Ten studies were based upon CHD, possibly due to CHDs being the most common birth defect (32). Traditional teaching of CHD has been delivered using 2D diagrams (for example, in textbooks), cross-sectional imaging modalities (MRI, CT, echocardiography), cadaveric specimens and off-the-shelf

Confidential: For Review Only

models (33). However, these tools pose a variety of problems, including difficulty in translating planar images into 3D reconstructions in the learner's mind, lack of tactile feedback in the case of imaging, and lack of availability of specific pathologies in the case of cadaveric specimens and plastic models. Studies focussing on CHD suggest that 3D models can overcome these issues, and have demonstrated increased understanding with more complex models (15). Congenital defects can be complex concepts to grasp (34) and therefore 3DP models have the potential to be a very useful educational tool to implement into routine practice. This is especially helpful for adult clinicians who will be involved in transition-to-adult services to provide care for the growing population of adults with congenital diseases (35,36). As CHD can be challenging for those with minimal training (34) it would be beneficial for future studies to assess the use of 3DP models for teaching non-paediatric clinicians.

When producing models for procedure simulation or teaching, the shape and size of the model should be considered, along with the suitability of the material used. Acrylonitrile butadiene styrene was shown to most accurately mimic bone when used with a drill and for craniotomy (13). However, replicating the consistency of soft tissue such as myocardium has proven difficult (10), and could detract from simulating the overall surgical experience. Another challenge posed is the lack of surrounding structures (e.g. the dura) and the inability to replicate bleeds or leaks (e.g. cerebrospinal fluid) (13). However, it has been demonstrated that soft tissues and artificial blood can be introduced into 3DP simulation models to replicate more closely in vivo anatomy and physiology (37).

Whilst here we focus specifically on paediatric teaching and training, other reviews exploring the use of 3DP models for undergraduate anatomy education and surgical training reported similar observations. Comparison of 3DP models to lectures, textbooks and 2D radiological imaging

Confidential: For Review Only

demonstrated a statistically significant improvement in medical student performance in anatomy tests (38–40). As a tool for surgical trainees, participants using 3DP models reported greater confidence in understanding surgical pathologies and intra-operative confidence in performing procedures (41,42). These systematic reviews also identified that many of these studies had non-randomised cohorts, small sample sizes and/or inconsistencies in measuring or reporting outcomes (such as differing pre- vs. post-test formatting or omission of certain measurements). These limitations are consistent with our findings and should prompt improvements in the design of future educational studies using 3DP in order to systematically and robustly further understand the role of the technology in teaching and training.

We also reviewed the cost of model production. Paediatric trainees in Low- and Middle-Income Countries (LMICs) can lack training opportunities due to limited traditional teaching resources (3,43). 3DP models may have a role in improving access to training resources in such countries. DeBoer and colleagues highlighted that their $2,500 3DP training model cost 43-250% less than other commercially available trainers at time of publication, with simple maintenance requirements (16). Using 3DP medical aids has shown benefit for patients in Sierra Leone (44); however, there is a paucity of evidence in the use of the technology in paediatric teaching and training in LMICs, which would warrant further investigation into its cost-effectiveness.

The current evidence base demonstrates a positive impact made by 3DP models on paediatric education and training – objectively and subjectively – for medical students and clinical trainees. The majority of studies failed to address sources of bias, assess clinically translatable outcomes, or evaluate long-term benefits. Future studies should recruit larger cohorts, ideally at a multi-centre level, randomise participants and adopt objective outcome measurements (e.g. anonymous anatomy examinations and monitoring of trainee clinical performance), including follow-up to assess

Confidential: For Review Only

long-term benefits. 3DP models also have the potential to be more sophisticated to simulate real scenarios including soft tissue, surrounding structures and blood.

References

1. Royal College of Paediatrics and Child Health. RCPCH position paper in response to the Shape of Training Review. 2017 Oct.
2. Bindal T, Wall D, Goodyear HM. Medical students' views on selecting paediatrics as a career choice. Eur J Pediatr. 2011;170(9).
3. Sohail AH, Maan MHA, Sachal M, Soban M. Challenges of training and delivery of pediatric surgical services in developing economies: a perspective from Pakistan. BMC Pediatr. 2019 Dec 16;19(1).
4. Al-Yassin A, Long A, Sharma S, May J. How can general paediatric training be optimised in highly specialised tertiary settings? Twelve tips from an interview-based study of trainees. BMJ Paediatr Open. 2017 Sep 4;1(1).
5. Hilliard R, Bannister SL, Amin H, Baird B. Paediatric medical education: Challenges and new developments. Paediatr Child Health. 2009 May 1;14(5).
6. Regitz-Zagrosek V, Roos-Hesselink JW, Bauersachs J, Blomström-Lundqvist C, Cífková R, De Bonis M, et al. 2018 ESC Guidelines for the management of cardiovascular diseases during pregnancy. Eur Heart J. 2018 Sep 7;39(34).
7. Lichtenberger JP, Tatum PS, Gada S, Wyn M, Ho VB, Liacouras P. Using 3D Printing (Additive Manufacturing) to Produce Low-Cost Simulation Models for Medical Training. Mil Med [Internet]. 2018 Mar 1;183(suppl_1):73–7. Available from: https://academic.oup.com/milmed/article/183/suppl_1/73/4959929
8. Ganguli A, Pagan-Diaz GJ, Grant L, Cvetkovic C, Bramlet M, Vozenilek J, et al. 3D printing for preoperative planning and surgical training: a review. Biomed Microdevices [Internet]. 2018

Confidential: For Review Only

Sep 4;20(3):65. Available from: http://link.springer.com/10.1007/s10544-018-0301-9

9. Giannopoulos AA, Mitsouras D, Yoo S-J, Liu PP, Chatzizisis YS, Rybicki FJ. Applications of 3D printing in cardiovascular diseases. Nat Rev Cardiol [Internet]. 2016 Dec 27;13(12):701–18. Available from: http://www.nature.com/articles/nrcardio.2016.170

10. Yoo SJ, Spray T, Austin EH, Yun TJ, van Arsdell GS. Hands-on surgical training of congenital heart surgery using 3-dimensional print models. J Thorac Cardiovasc Surg [Internet]. 2017;153(6):1530–40. Available from: http://dx.doi.org/10.1016/j.jtcvs.2016.12.054

11. Moher D, Liberati A, Tetzlaff J, Altman DG. Preferred Reporting Items for Systematic Reviews and Meta-Analyses: The PRISMA Statement. PLoS Med [Internet]. 2009 Jul 21;6(7):e1000097. Available from: https://dx.plos.org/10.1371/journal.pmed.1000097

12. Beller EM, Glasziou PP, Altman DG, Hopewell S, Bastian H, Chalmers I, et al. PRISMA for Abstracts: Reporting Systematic Reviews in Journal and Conference Abstracts. PLoS Med [Internet]. 2013 Apr 9;10(4):e1001419. Available from: https://dx.plos.org/10.1371/journal.pmed.1001419

13. Cheng D, Yuan M, Perera I, O'Connor A, Evins AI, Imahiyerobo T, et al. Developing a 3D composite training model for cranial remodeling. J Neurosurg Pediatr. 2019;24(6):632–41.

14. Su W, Xiao Y, He S, Huang P, Deng X. Three-dimensional printing models in congenital heart disease education for medical students: A controlled comparative study. BMC Med Educ. 2018;18(1):1–6.

15. Smerling J, Marboe CC, Lefkowitch JH, Pavlicova M, Bacha E, Einstein AJ, et al. Utility of 3D Printed Cardiac Models for Medical Student Education in Congenital Heart Disease: Across a Spectrum of Disease Severity. Pediatr Cardiol [Internet]. 2019;40(6):1258–65. Available from: https://doi.org/10.1007/s00246-019-02146-8

16. Deboer EM, Wagner J, Kroehl ME, Albietz J, Shandas R, Deterding RR, et al. Three-Dimensional Printed Pediatric Airway Model Improves Novice Learners' Flexible Bronchoscopy Skills with Minimal Direct Teaching from Faculty. Simul Healthc.

Confidential: For Review Only

2018;13(4):284–8.

17. Cromeens BP, Ray WC, Hoehne B, Abayneh F, Adler B, Besner GE. Facilitating surgeon understanding of complex anatomy using a three-dimensional printed model. J Surg Res [Internet]. 2017;216:18–25. Available from: http://dx.doi.org/10.1016/j.jss.2017.04.003

18. Costello JP, Olivieri LJ, Su L, Krieger A, Alfares F, Thabit O, et al. Incorporating three-dimensional printing into a simulation-based congenital heart disease and critical care training curriculum for resident physicians. Congenit Heart Dis. 2015;10(2):185–90.

19. Gasior AC, Reck C, Lane V, Wood RJ, Patterson J, Strouse R, et al. Transcending Dimensions: a Comparative Analysis of Cloaca Imaging in Advancing the Surgeon's Understanding of Complex Anatomy. J Digit Imaging. 2019;32(5):761–5.

20. Biglino G, Capelli C, Koniordou D, Robertshaw D, Leaver LK, Schievano S, et al. Use of 3D models of congenital heart disease as an education tool for cardiac nurses. Congenit Heart Dis. 2017;12(1):113–8.

21. D. P. Utility of 3 dimensional printing of hearts with complex congenital heart disease in the education of pediatric trainees and sonographers. FASEB J [Internet]. 2016;30(Meeting Abstracts):5–7. Available from: http://www.fasebj.org/content/30/1_Supplement/787.7.abstract?sid=a4993bad-734d-447f-a363-e524a7c6dee2%0Ahttp://ovidsp.ovid.com/ovidweb.cgi?T=JS&PAGE=reference&D=emed17&NEWS=N&AN=72317314

22. Costello JP, Olivieri LJ, Krieger A, Thabit O, Marshall MB, Yoo SJ, et al. Utilizing Three-Dimensional Printing Technology to Assess the Feasibility of High-Fidelity Synthetic Ventricular Septal Defect Models for Simulation in Medical Education. World J Pediatr Congenit Hear Surg. 2014;5(3):421–6.

23. Hadden R, Grover S, Chuen J C-BJ. Assessing the Utility of 3D Printed Models of Mullerian Tract Anomalies for Clinical Education. Aust NEW Zeal J Obstet Gynaecol. 2018;58:55.

Confidential: For Review Only

24. Loke YH, Harahsheh AS, Krieger A, Olivieri LJ. Usage of 3D models of tetralogy of Fallot for medical education: Impact on learning congenital heart disease. BMC Med Educ. 2017;17(1):1–8.

25. Jones TW, Seckeler MD. Use of 3D models of vascular rings and slings to improve resident education. Congenit Heart Dis. 2017;12(5):578–82.

26. White SC, Sedler J, Jones TW, Seckeler M. Utility of three-dimensional models in resident education on simple and complex intracardiac congenital heart defects. Congenit Heart Dis. 2018;13(6):1045–9.

27. Reed DA, Cook DA, Beckman TJ, Levine RB, Kern DE, Wright SM. Association Between Funding and Quality of Published Medical Education Research. JAMA [Internet]. 2007 Sep 5;298(9):1002. Available from: http://jama.jamanetwork.com/article.aspx?doi=10.1001/jama.298.9.1002

28. Zhang J, Zhang FZ, Hao JS, Chen M, Zheng J, Ni X. [The application of 3D simulationtemporal-bone-system for temporal bone anatomy training]. Lin Chung Er Bi Yan Hou Tou Jing Wai Ke Za Zhi [Internet]. 2016 Aug 20;30(16):1325–8. Available from: http://www.ncbi.nlm.nih.gov/pubmed/29797982

29. Zendejas B, Brydges R, Wang AT, Cook DA. Patient outcomes in simulation-based medical education: A systematic review. Journal of General Internal Medicine. 2013.

30. Griswold-Theodorson S, Ponnuru S, Dong C, Szyld D, Reed T, McGaghie WC. Beyond the Simulation Laboratory. Acad Med [Internet]. 2015 Nov;90(11):1553–60. Available from: http://journals.lww.com/00001888-201511000-00032

31. Tack P, Victor J, Gemmel P, Annemans L. 3D-printing techniques in a medical setting: a systematic literature review. Biomed Eng Online [Internet]. 2016 Dec 21;15(1):115. Available from: http://biomedical-engineering-online.biomedcentral.com/articles/10.1186/s12938-016-0236-4

32. van der Linde D, Konings EEM, Slager MA, Witsenburg M, Helbing WA, Takkenberg JJM, et al.

Confidential: For Review Only

Birth Prevalence of Congenital Heart Disease Worldwide. J Am Coll Cardiol [Internet]. 2011 Nov;58(21):2241–7. Available from: https://linkinghub.elsevier.com/retrieve/pii/S0735109711030798

33. Seslar SP, Shepard CW, Giroud JM, Aiello VD, Cook AC, Spicer DE, et al. Lost treasures: a plea for the systematic preservation of cadaveric heart specimens through three-dimensional digital imaging. Cardiol Young [Internet]. 2015 Dec 16;25(8):1457–9. Available from: https://www.cambridge.org/core/product/identifier/S1047951115002589/type/journal_article

34. Webb CL, Jenkins KJ, Karpawich PP, Bolger AF, Donner RM, Allen HD, et al. Collaborative Care for Adults With Congenital Heart Disease. Circulation [Internet]. 2002 May 14;105(19):2318–23. Available from: https://www.ahajournals.org/doi/10.1161/01.CIR.0000017557.24261.A7

35. Viner RM. Transition of care from paediatric to adult services: one part of improved health services for adolescents: Figure 1. Arch Dis Child [Internet]. 2008 Feb;93(2):160–3. Available from: https://adc.bmj.com/lookup/doi/10.1136/adc.2006.103721

36. Marelli AJ, Mackie AS, Ionescu-Ittu R, Rahme E, Pilote L. Congenital Heart Disease in the General Population. Circulation [Internet]. 2007 Jan 16;115(2):163–72. Available from: https://www.ahajournals.org/doi/10.1161/CIRCULATIONAHA.106.627224

37. O’Reilly MK, Reese S, Herlihy T, Geoghegan T, Cantwell CP, Feeney RNM, et al. Fabrication and assessment of 3D printed anatomical models of the lower limb for anatomical teaching and femoral vessel access training in medicine. Anat Sci Educ [Internet]. 2016 Jan;9(1):71–9. Available from: http://doi.wiley.com/10.1002/ase.1538

38. Yuen J. What Is the Role of 3D Printing in Undergraduate Anatomy Education? A Scoping Review of Current Literature and Recommendations. Med Sci Educ [Internet]. 2020 Sep 3;30(3):1321–9. Available from: http://link.springer.com/10.1007/s40670-020-00990-5

39. Fleming C, Sadaghiani MS, Stellon MA, Javan R. Effectiveness of Three-Dimensionally Printed Models in Anatomy Education for Medical Students and Resident Physicians: Systematic

Confidential: For Review Only

Review and Meta-Analysis. J Am Coll Radiol [Internet]. 2020 Oct;17(10):1220–9. Available from: https://linkinghub.elsevier.com/retrieve/pii/S1546144020305561

40. Ye Z, Dun A, Jiang H, Nie C, Zhao S, Wang T, et al. The role of 3D printed models in the teaching of human anatomy: a systematic review and meta-analysis. BMC Med Educ [Internet]. 2020 Dec 29;20(1):335. Available from: https://bmcmededuc.biomedcentral.com/articles/10.1186/s12909-020-02242-x

41. Langridge B, Momin S, Coumbe B, Woin E, Griffin M, Butler P. Systematic Review of the Use of 3-Dimensional Printing in Surgical Teaching and Assessment. J Surg Educ [Internet]. 2018 Jan;75(1):209–21. Available from: https://linkinghub.elsevier.com/retrieve/pii/S1931720417301836

42. Vakharia VN, Vakharia NN, Hill CS. Review of 3-Dimensional Printing on Cranial Neurosurgery Simulation Training. World Neurosurg [Internet]. 2016 Apr;88:188–98. Available from: https://linkinghub.elsevier.com/retrieve/pii/S1878875015017350

43. Cass H, Barclay S, Gerada C, Lumsden DE, Sritharan K. Complexity and challenge in paediatrics: a roadmap for supporting clinical staff and families. Arch Dis Child [Internet]. 2019 Jun 11;archdischild-2018-315818. Available from: https://adc.bmj.com/lookup/doi/10.1136/archdischild-2018-315818

44. van der Stelt M, Verhulst AC, Vas Nunes JH, Koroma TAR, Nolet WWE, Slump CH, et al. Improving Lives in Three Dimensions: The Feasibility of 3D Printing for Creating Personalized Medical Aids in a Rural Area of Sierra Leone. Am J Trop Med Hyg [Internet]. 2020 Apr 1;102(4):905–9. Available from: http://www.ajtmh.org/content/journals/10.4269/ajtmh.19-0359

Funding statement

Confidential: For Review Only

The authors received no specific grant from any funding agency in the public, commercial or not-for-profit sectors for conducting this review. The authors acknowledge the generous support of the British Heart Foundation (CH/17/1/32804) and The Grand Appeal (Bristol Children's Hospital Charity).

Competing interests statement

The authors declare that the research was conducted in the absence of any commercial or financial relationships that could be construed as a potential conflict of interest.

Patient consent for publication

Not required.

Ethics approval

Ethical approval was not required as no primary patient data was used in this review.

Data availability statement

All data relevant to the study are included in the article or uploaded as supplemental information. No additional information.

Author contributions

Confidential: For Review Only

Ashar Asif conceived the study and drafted the study protocol, registered the review to Prospero, conducted the review searches, was first reviewer of the eligible studies and lead the writing of the manuscript.

Elgin Meng Lee reviewed eligible papers and contributed to the writing of the manuscript.

Massimo Caputo assisted in editing the final manuscript.

Giovanni Biglino assisted in editing the final manuscript.

Andrew Shearn conceived and supervised the study, was adjudicator for any conflicts in study selection for the review, and assisted in editing the final manuscript.

All authors have read and approved the final manuscript.

Confidential: For Review Only

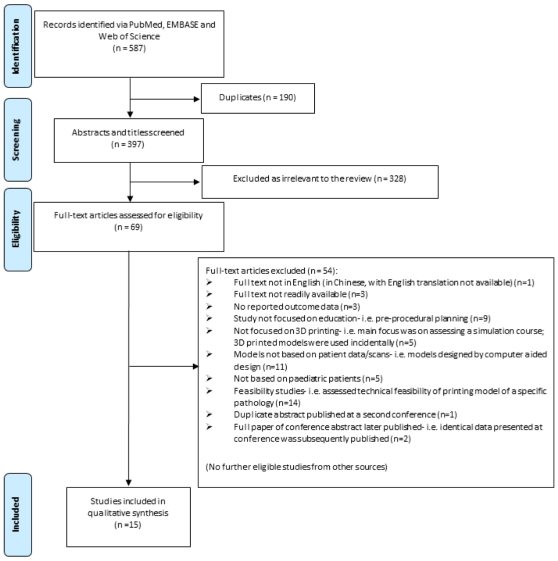

Figure 1. PRISMA flowchart of identifying eligible studies.

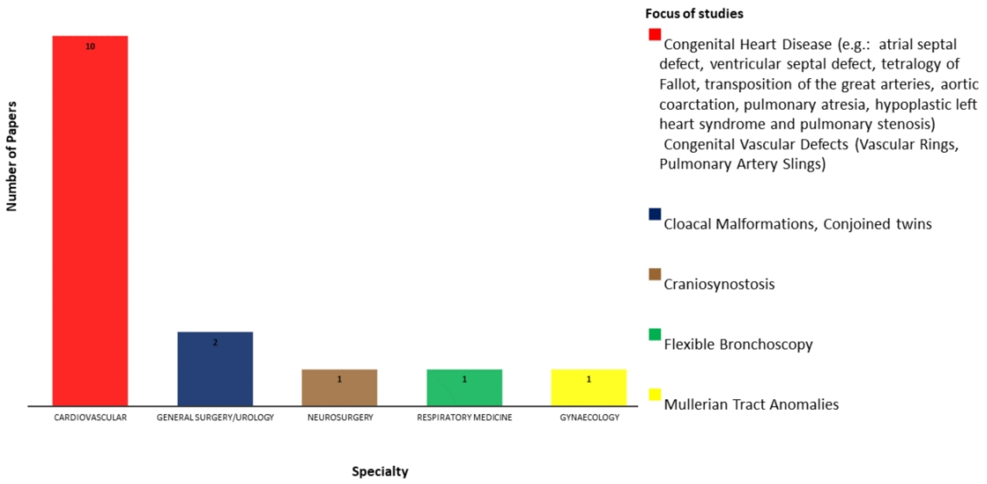

Figure 2. Breakdown of specialties for included studies
